# Vascular Proteomics Reveal Novel Proteins Involved in SMC Phenotypic Change: OLR1 as a SMC Receptor Regulating Proliferation and Inflammatory Response

**DOI:** 10.1371/journal.pone.0133845

**Published:** 2015-08-25

**Authors:** Dong Hoon Kang, Mina Choi, Soyoung Chang, Min Young Lee, Doo Jae Lee, Kyungsun Choi, Junseong Park, Eun Chun Han, Daehee Hwang, Kihwan Kwon, Hanjoong Jo, Chulhee Choi, Sang Won Kang

**Affiliations:** 1 Department of Life Science and Research Center for Cell Homeostasis, Ewha Womans University, Seoul 120–750, Korea; 2 Department of Cardiology and College of Medicine, Ewha Womans University, Seoul 120–750, Korea; 3 Global Top5 Research program, Ewha Womans University, Seoul 120–750, Korea; 4 Department of Bio and Brain Engineering, KAIST, Daejeon 305–701, Korea; 5 School of Interdisciplinary Bioscience and Bioengineering, POSTECH, Pohang 790–784, Korea; 6 Department of New Biology and Center for Plant Aging Research, Institute for Basic Science, DGIST, Daegu 711–873, Korea; 7 Wallace H. Coulter Department of Biomedical Engineering, Georgia Institute of Technology and Emory University, Atlanta, GA30322, United States of America; William Harvey Research Institute, Barts and The London School of Medicine and Dentistry, Queen Mary University of London, UNITED KINGDOM

## Abstract

Neointimal hyperplasia of vascular smooth muscle cells (VSMC) plays a critical role in atherosclerotic plaque formation and in-stent restenosis, but the underlying mechanisms are still incompletely understood. We performed a proteomics study to identify novel signaling molecules organizing the VSMC hyperplasia. The differential proteomics analysis in a balloon-induced injury model of rat carotid artery revealed that the expressions of 44 proteins are changed within 3 days post injury. The combination of cellular function assays and a protein network analysis further demonstrated that 27 out of 44 proteins constitute key signaling networks orchestrating the phenotypic change of VSMC from contractile to epithelial-like synthetic. Among the list of proteins, the *in vivo* validation specifically revealed that six proteins (Rab15, ITR, OLR1, PDHβ, PTPε) are positive regulators for VSMC hyperplasia. In particular, the OLR1 played dual roles in the VSMC hyperplasia by directly mediating oxidized LDL-induced monocyte adhesion via NF-κB activation and by assisting the PDGF-induced proliferation/migration. Importantly, OLR1 and PDGFRβ were associated in close proximity in the plasma membrane. Thus, this study elicits the protein network organizing the phenotypic change of VSMC in the vascular injury diseases such as atherosclerosis and discovers OLR1 as a novel molecular link between the proliferative and inflammatory responses of VSMCs.

## Introduction

Atherosclerosis is an inflammatory vascular disease accompanying with the occlusion of the arterial vessels. The early atherosclerotic lesions include sub-endothelial accumulations of lipid-engorged macrophages (foam cells), which are represented as ‘fatty streak’ [1]. Fatty streaks are not clinically harmful, but they can be the precursors of more advanced fibrous and plaque-type lesions characterized by the accumulation of lipid-rich necrotic debris and vascular smooth muscle cells (VSMCs). During the disease progression, the arterial wall gradually thickens and hardens by the formation of atherosclerotic plaques. If the plaques become fragile, they abruptly rupture and often cause myocardial infarction or stroke [2]. Consequently, atherosclerosis progresses for many decades and cannot easily be recognized before the appearance of ischemic symptoms. Once the vascular narrowing is diagnosed, the way to eliminate the plaques and dilate the occluded vessel area is the vascular intervention by percutaneous transluminal angioplasty. However, the balloon-induced injury to the arterial endothelium triggers the thrombotic activation of VSMC de-differentiation and hence results in the dysplastic neointimal thickening, so-called in-stent restenosis. Such an in-stent restenosis has been overcome by using cytotoxic drug-eluting stents but it is still responsible for the clinical failures of vascular interventions [3, 4]. Overall, the SMC hyperproliferation is the key step in the formation of both atherosclerotic plaques and neointimal thickening. Given that the SMC hyperproliferation is triggered by multiple factors including the pro-inflammatory factors [5–8], it is important to understand the complex cellular and molecular communication networks orchestrating the hyperproliferation process.

Many attempts have been undertaken to identify the early diagnostic biomarkers and therapeutic targets for atherosclerosis in human samples or model animals [9]. For example, the clinical data from a follow-up study of 3,209 participants in the Framingham Heart Study for more than 7 years revealed several valuable biomarkers such as C-reactive protein, B-type natriuretic peptide, renin, urinary albumin, homocysteine [10]. Plasma proteomics and metabolomics experiments have also been aggressively conducted for many years to search for circulating biomarkers that show a correlation with the atherosclerosis progression [11, 12]. Furthermore, an enormous number of studies have focused on therapeutic targets for atherosclerosis. Some of targets include paraoxonase [13], platelet-derived growth factor (PDGF) [7], Fas ligand (CD95/Apo-1) [14], fatty acid-binding protein-4 [15], JNK2 [16], and netrin-1 [17]. Nonetheless, the above studies have described the problems owing to the inter-individual variability and inaccessibility of human tissues with vascular thickening. Therefore, we adapted a proteomic strategy using the balloon-injured arterial vessels of genetically congenic rats and validated the proteomes in human aortic VSMCs to search for the human-relevant candidates. As results, we performed the quantitative and reproducible proteome analyses of more than 2,100 protein spots and identified nine novel candidates that positively regulate the VSMC activities related to hyperproliferation. In particular, we determined a novel signaling role of the oxidized LDL receptor-1 (OLR1) among the validated candidates in VSMCs.

## Materials and Methods

### Materials

Human aortic SMCs (HASMC) were purchased from Clonetics-Bio Whittaker (Venders, Belgium) and grown on 0.1% gelatin-coated plate at 37°C in a humidified incubator containing 5% CO_2_ in Smooth Muscle Cell Basal Medium (SmBM) SingleQuotes with 10% fetal bovine serum (FBS) and full supplements (Clonetics-BioWhittaker; Cat no. cc-4149). They were used for the study at passage number 7 showing active growth. The human monocytic cell line U937 was obtained from American Type Culture Collection (Rockville, MD) and maintained in culture medium RPMI 1640 (WelGENE lnc) supplemented with 10% FBS (HyClone).

For quantitative real-time PCR (qPCR), the primers specific for rat Rab15 (QT01614088), rat ITR (QT01608635), rat OLR1 (QT00191254), rat PDHB (QT00431263), rat PTPRE (QT02335606), rat UCHL1 (QT01081080), rat PRKACA (QT01588629), and rat VDAC1 (QT00190561) were purchased from Qiagen. Primers for rat β-actin reference gene were (sense) 5’- TGGATCAGCAAGCAGGAGTAT-3’ and (antisense) 5’-GCATTTGCGGTGGCAGAT3’. Primers for rat 18S rRNA reference gene were (sense) 5’-GGCCGTTCTTAGTTGGTGGAGCG-3’ and (antisense) 5’-CTGAACGCCACTTGTCCCTC-3’.

Anti-tubulin antibody was purchased from Sigma-Aldrich. Anti-PDI, enolase-1, Lamin B, ERK2, GAPDH, IκBα (C-21), PDGFR-β (M-20) and VDAC1 antibodies were purchased from Santa Cruz Biotechnology. Anti-PTPε antibodies for human immunohistochemistry staining were purchased from Atlas Antibodies (Stockholm, Sweden). Anti-PCNA, β-actin, ERK (pT202/pY204) and IκBα (pSer32/36) antibodies were purchased from Cell Signaling Technology. Anti-phosphotyrosine (4G10) and UCHL1 antibodies and PDGF-BB were from Upstate Biotechnology. Anti-OLR1 and transferrin receptor antibodies were purchased from R&D systems. Anti-CD18 antibody was purchased from BD Bioscience. Rabbit polyclonal antibody against PDGFRβ (pY857) was produced as previously described [18].

Minimally oxidized low-density lipoproteins (mmLDLs) were purchased from KALEN Biomedical (Maryland, USA). DuoLink In Situ Fluorescence reagents were purchased from Sigma-Aldrich.

### Animal study

Animal experiments using Sprague-Dawley rats were granted by approval of the Institutional Animal Care and Use Committee (IACUC) of Ewha Womans University and conformed to the Guide for Care and Use of Laboratory Animals published by the US National Institutes of Health (The National Academies Press, 8^th^ Edition, 2011). All surgical procedures were performed under inhalational anesthesia with isoflurane gas (N_2_O:O_2_/70%:30%), and all efforts were made to minimize suffering. The ten-week-old male Sprague-Dawley rats (Charles River, U.S.A.) were for used for a balloon-induced carotid injury model. The animal experiments for main and supplementary figures were repeated twice. The detailed animal numbers in experimental groups are described in the figure legends.

### Balloon-induced injury of rat carotid artery

The ten-week-old male Sprague-Dawley rats were used for a balloon-induced injury model of rat carotid artery. A balloon injury was created using an infiltrated 2F Fogarty balloon embolectomy catheter in the left common carotid artery, as previously described [19]: The rats were anaesthetized by inhalation of isoflurane gas (N_2_O:O_2_/70%:30%); the left external carotid artery was exposed; and its branches were electro-coagulated. A catheter was pushed 1 cm through the transverse arteriotomy of the external carotid artery, and the endothelial denudation was achieved by three passes along the common carotid artery. After removal of the catheter, the punched area was sealed and the clapped common carotid artery was opened to resume the blood flow. After the surgical operation, the rats were recovered in the cages for various times (zero, 0.75, 3, 5, and 7 days for proteomics study; 10 days for *in vivo* validation of selected proteins). The sham operation was used as a control for zero time.

### Subcellular fractionation of carotid arteries

After rats were anaesthetized by inhalation of isoflurane gas (N_2_O:O_2_/70%:30%) and perfused with heparinized saline, the balloon-injured left carotid arteries were removed. The arteries (8 rats per group) were immediately washed in cold saline to remove erythrocytes and then minced with a razor. If necessary, the excess blood was flushed out from the lumen area of arteries by 26 gauge syringe. The minced carotid arteries were resuspended in 1 ml of hypotonic buffer (10 mM HEPES pH 7.5, 2 mM MgCl_2_, 25 mM KCl, 0.5 mM EDTA, 0.5 mM EGTA, 1 mM DTT, 1 mM AEBSF, 5 μg/ml Leupeptin and Aprotinin, 1 mM Na_3_VO_4_, 5 mM NaF) and placed on ice for 30 min. Arteries were gently homogenized with 25 strokes in Dounce homogenizer and the homogenates were centrifuges twice at 750 x g for 10 min. The first pellets were kept on ice for nuclei preparation. The supernatants were further centrifuged at 100,000 x g for 1 hr with a table top ultracentrifuge (Beckman Coulter). The resulting second pellet and supernatant (S-100) were designated as heavy/light membrane and cytosolic fractions, respectively. For isolation of pure nuclei, the first pellets were resuspended in 1 ml detergent-containing hypotonic buffer (10 mM Hepes pH 7.5, 2 mM MgCl_2_, 10 mM KCl, 0.2% NP-40, 0.5 mM EDTA, 0.5 mM EGTA, 1 mM AEBSF, 5μg/ml Leupeptin and Aprotinin, 1 mM Na_3_VO_4_, 5 mM NaF) and then disrupted by passing 10 times through 22-guage needle. After repeating this process three times, the resulting pellets were designated as nuclei fraction. The S-100 cytosolic fraction was concentrated by TCA precipitation. The protein precipitates were rinsed with acetone twice to remove residual TCA solution and subjected to the proteome analyses. Finally, the protein-containing pellets of the separated fractions were dissolved in a lysis solution containing 8 M urea, 4% CHAPS, 2% IPG buffer, and protease inhibitor and subjected to the following 2D-DIGE analyses. The protein concentration was determined by a Bradford assay.

### Two dimensional difference gel electrophoresis (2D-DIGE)

The two consecutive samples among 5 serial time points were differently labeled with the *N*-hydroxysuccinimidyl ester forms of Cy3 and Cy5 fluorescent dyes in a pair. The equal amount of protein (10 μg) from five serial samples were pooled and labeled with Cy2 fluorescent dye as an internal standard. For dye labeling, 50 μg of protein was resuspended in 15~20 μl of labeling buffer (7 M urea, 2 M thiourea, 4% CHAPS, 30 mM Tris, pH 8.5) and reacted with 400 pmol of each dye for 30 min on ice in the dark. The reaction was then quenched with the addition of 10 mM lysine (1μl for each reaction). The Cy3-labeled sample (50 μg), Cy5-labeled sample (50 μg), and the Cy2-labeled internal standard (50 μg) were combined and mixed with an equal volume of 2 × rehydration buffer (7 M urea, 2 M thiourea, 4% CHAPS, 130 mM DTT, 2% pH 4–7 IPG buffer or pH 3–10 NL IPG buffer) in a final volume of 120 μl. Then, the samples were subjected to the first-dimensional isoelectrofocusing on 13-cm pH 4–7 or pH 3–10 NL IPG strips (Amersham Biosciences/GE Healthcare). The second-dimension was performed on 10% polyacrylamide denaturing gels.

The fluorescence images of DIGE gels were taken by a Typhoon 9400 variable mode imager with at 100-μm resolution (Amersham Biosciences/GE Healthcare) and analyzed with the DeCyder version 6.5 suite of software tools (Amersham Biosciences/GE Healthcare). After the fluorescence analysis, the 2D-DIGE gels were silver-stained and merged with the corresponding fluorescence images for excision of protein spots showing differential expression. The excised spots were subjected to the mass spectrometry following in-gel trypsin digestion as previously described [20].

### Mass spectrometry

The identification of protein spots was performed by MALDI-TOF or ESI-MS/MS. For MALDI-TOF, the tryptic digests were reconstituted in 10 μl of 0.1% trifluoroacetic acid and treated with ZipTips containing a C_18_ resin (Millipore) according to the manufacturer's instructions. The peptides were dissolved in 2 μl of matrix solution (1% w/v of α-cyano-4-hydroxycinnamic acid (CHCA) in 50% acetonitrile, 0.1% v/v TFA) and the aliquots (0.5 μl) were applied twice to a target disk and dried. Mass Spectra were obtained using an Applied Biosystems Voyager-DE^TM^ STR workstation (Foster City, CA, USA). The raw spectrum were opened in Voyager Data Explorer software (version 4.6) and calibrated with two peptides resulting from trypsin autolysis (m/z 842.5100 and 2211.1046). Monoisotopic peptide masses only were assigned for searching, the peak detection threshold was manually adjusted over the background, and the peak list was copied to Mascot public interface and searched against rat SwissProt database (version 49.0, released February 7, 2006, 17548 entries) using the following parameters: trypsin as digest enzyme, two possible missed cleavages, acetylation (K), pyroglu (N-term E, Q), oxidation (M), phosphorylation (S,T,Y), propionamidation or cabamidomethylation (C) as variable modification, and peptide tolerance of 20 ppm. Results were scored using probability-based Mowse score (Protein score is -10 × log(*p*) where *p* is the probability that the observed match is a random event. The protein score greater than 50 was determined to be significant (*p* <0.05).

The MS/MS analysis for peptide sequencing was done in nano flow reversed-phased HPLC/ESI/MS with a mass spectrometer (Q-TOF Ultima global, Waters Co. UK). The tryptic digests (5 μl) were dissolved in buffer A (water/ACN/formic acid; 95:5:0.2, v/v) and injected onto a C_18_ reversed-phase 75 μm i. d. x 150 mm analytical column (3 μm particle size, Atlantis dC18, Waters) with an integrated electrospray ionization SilicaTip (± 10 μm, New Objective, USA). The peptides were desalted in a line prior to separation using a trap column (i.d. 0.35 x 50 mm, OPTI-PAK C_18_, Waters) cartridge and eluted by a linear gradient of 5–80% buffer B (water/ACN/formic acid; 5:95:0.2, v/v) over 120 min. The flow rate was set to 200 nl/min by a split/splitless inlet and the capillary voltage (3.0 kV) was applied to the HPLC mobile phase before spraying. Chromatography was performed online using the instrument’s control software MassLinx of Q-TOF Ultima global (Version 4.0, Waters Co. U.K.). The mass spectrometer was programmed to record scan cycles composed of one MS scan followed by MS/MS scans of the three most abundant ions in each MS scan within 10s window. The acquired spectra were automatically processed using ProteinLynx Global Server data processing software (version 2.1, Waters Co.UK) and matched against amino acid sequences in rat SwissProt database (version 50.8. released September 11, 2008, 21455 entries) using Mascot search (version 2.1.03 Matrix Science, London, UK) and MODi (Korea, http://modi.uos.ac.kr/modi/). The search parameters were as follow: 0.3 Da tolerance for peptide and fragment ions; digestion with trypsin with up to two missed cleavage allowed, acetylation (K), deamidation (N,Q), methylation (K), dimethylation (K), pyroglu (N-term E, Q), oxidation (M), phosphorylation (S,T,Y), ubiquitination (K), propionamidation or cabamidomethylation (C) were set as a variable modification but not fixed modification. Results were scored using probability-based Mowse score (Protein score is -10 × log(*p*) where *p* is the probability that the observed match is a randem event. The protein score greater than 50 was determined to be significant (*p*<0.05). In addition, a minimum total score of 50, comprising at least a peptide match of ion score more than 20, was arbitrarily set as threshold for acceptance. All reported assignments were verified by automatic and manual interpretation of spectra.

### Western blotting

The protein content of tissue or cell extracts was quantified using the Bradford reagent (Bio-Rad). The samples were mixed with 5x Laemmli sample buffer and boiled for 5 min. The boiled samples (15 μg protein) were separated on 10% SDS polyacrylamide gels and then transferred to nitrocellulose membranes. The membranes were incubated with a blocking buffer (5% nonfat milk or 1% BSA in Tris-buffered saline containing 0.5% Tween-20) for 1 hr with rocking at room temperature. The blots were incubated with the indicated primary antibodies in the blocking buffer at 4°C overnight. After washing three times with TBST, the blots were incubated with horseradish peroxidase-conjugated sheep anti-mouse immunoglobulin G (IgG; 1:5000) or with goat anti-rabbit IgG (1:3000) for 1 hr at room temperature. The specific immune-reactive protein bands were visualized using Amersham ECL detection system (GE healthcare).

### Proliferation and migration assays

For proliferation assay, the HASMCs were seeded at a density of 4000 cells/well in a final volume of 100 μl onto 96-well plates containing siRNA-siPORT NeoFX reagent premixes (reverse transfection, Ambion). After a 24-hr transfection, the cells were serum-starved for 18 hr, and then stimulated with PDGF-BB (25 ng/ml, Upstate Biotenology Inc) in the SmBM basal medium for an additional 24 hr. The extent of cell proliferation was measured using a WST-1 cell proliferation assay kit (Roche Diagnostics, USA). The cell number was expressed as light absorbance at 450 nm, which was averaged from triplicate wells after subtracting the turbidity at 600 nm.

The migration assay was performed in 24-well Transwell culture chambers (Costar; 8-μm pore size). The bottom of the filter was coated with gelatin B (1 mg/ml) and air-dried for 1 hr. HASMCs (6 × 10^3^ cells/ml) were added to the upper chambers, which contained transfection premixes. After a 24-hr transfection, the transfected cells were serum-starved overnight. The SmBM basal media containing PDGF-BB (25 ng/ml) and 0.5% BSA were added to the bottom chambers. The upper chamber wells were filled with SmBM basal media containing 0.5% BSA. Transwell chambers were incubated at 37°C/5% CO_2_ for 8 hr. After incubation, the non-migrated cells were removed from the top of the filters, and the cells that migrated onto the bottom of filters were fixed and stained with 0.6% hematoxylin and 0.5% eosin. The stained cells were photographed and counted. The number of migrating cells was averaged from triplicate wells.

### Monocyte adhesion assay

U937 monocytes (1×10^6^ cells/ml) were incubated in the presence of IFN-γ (50 μg/ml) and LPS (100 ng/ml) for 24 hr. Separately, HASMCs were seeded on at a density of 2×10^3^ cells/well in a final volume of 100 μl into the 96-well plates containing 20 μl transfection premixes as described in the proliferation assay. After 48 hr, the cells were stimulated with OxLDL (100 μg/ml) or TNF-α (10 ng/ml) for further 24 hr. U937 cells were labeled with 4 μM tetramethylrhodamine ethyl ester, perchlorate (T-669, Molecular Probes, Eugene, OR) for 30 min. The labeled U937 cells (1×10^5^ cells/well) were added on stimulated HASMC and incubated for 1 hr. Non-adherent cells were removed by washing three times with PBS. The adherent U937 cells on the HASMC surface were identified in 2 randomly-selected microscopic areas under inverted microscope (Axiovert 200 Basic standard, Zeiss, Germany) and then counted by Phoretix 2D Evolution (Nonlinear Dynamics Ltd, USA)

### Network analysis

Two networks were first generated for the 25 proteins using 1) ‘Manual Expand’ (ME) algorithm with the two maximum number of interactions and 2) ‘Direct Interaction’ (DI) algorithm in MetaCore(ver 6.13) [21]. These two networks were then combined to generate an initial network model. To clarify the relationships among the 25 proteins, the nodes not contributing to functional connections between the 25 proteins were removed from the network model as follows. We identified gene ontology biological processes (GOBPs) significantly (*P*<0.01) represented by the proteins in the initial network using DAVID software [22] and GO Trimming (ver 2.0) [23] that removes redundant GOBPs. We then pruned the nodes not involved in these GOBPs. Also, among the proteins in the same protein family, we pruned the proteins that do not interact with the 25 protein seeds. After computing the number of interactions with the 25 proteins (K) for every node, we pruned transcription factors with K<5. The resulting final network was visualized using Cytoscape (v. 2.8.1) [24]. The nodes involved in similar or same GOBPs and KEGG pathways were grouped into the same modules, each of which was named by the corresponding GOBP or KEGG pathway.

### Catheter-mediated intramural delivery of siRNAs into carotid artery

The rat-specific siRNA SMART pools (Thermo Scientific Dharmacon, 200 nM) were premixed with siPORT^TM^ NeoFX reagent following the manufacturer’s instructions (Ambion). The common carotid arteries were balloon-injured and briefly washed with Opti-MEM. The transfection pre-mix (200 μl) was then administered through the catheter with punctured balloon [19, 25]. The vessel was incubated for 15 min for the efficient transfection and then ligated. A fluorescent dye-conjugated control siRNA (siGLO-Red, Dharmacon) was used for optimizing the intramural transfection efficiency.

### Histological analysis

Rats were anesthetized and the common carotid arteries were excised after transcardiac perfusion-fixation with heparinized saline containing 3.7% formaldehyde. The vessels were paraffin embedded and sectioned by rotary microtome (Leica RM2255). The two serial tissue sections (4 μm in thickness) were obtained from the middle area of common carotid arteries and stained with haematoxylin and eosin (HE). The luminal, internal elastic laminal, and external elastic laminal areas were measured using NIH Image v1.62. The intimal and medial areas were determined by subtraction of the luminal area from the internal elastic area and by subtraction of the internal elastic area from the external elastic area. The values from two serial sections per rat were averaged for analysis.

### Preparation of normal arterial vessel sample

With informed consent, human mammary arteries were harvested from a patient who underwent a mastectomy in accordance with the guidelines of the Committee on Ethics of Ewha womans University. The isolated artery tissues were immediately embedded in OCT medium and frozen at -80°C. The frozen tissue sections (7 μm in thickness) were prepared and used as normal arterial control for immunohistochemistry.

### Statistical analysis

Data were analyzed with either Student t-test for comparisons between two groups or one-way ANOVA with Tukey’s ‘honestly significant difference’ post hoc test for multiple groups (SPSS 12.0K for Windows, SPSS, Chicago, IL, USA). A *P* < 0.05 was considered to be statistically significant.

## Results

### Balloon-induced injury induces a significant proteome change in rat carotid vessels

**Fig 1:**
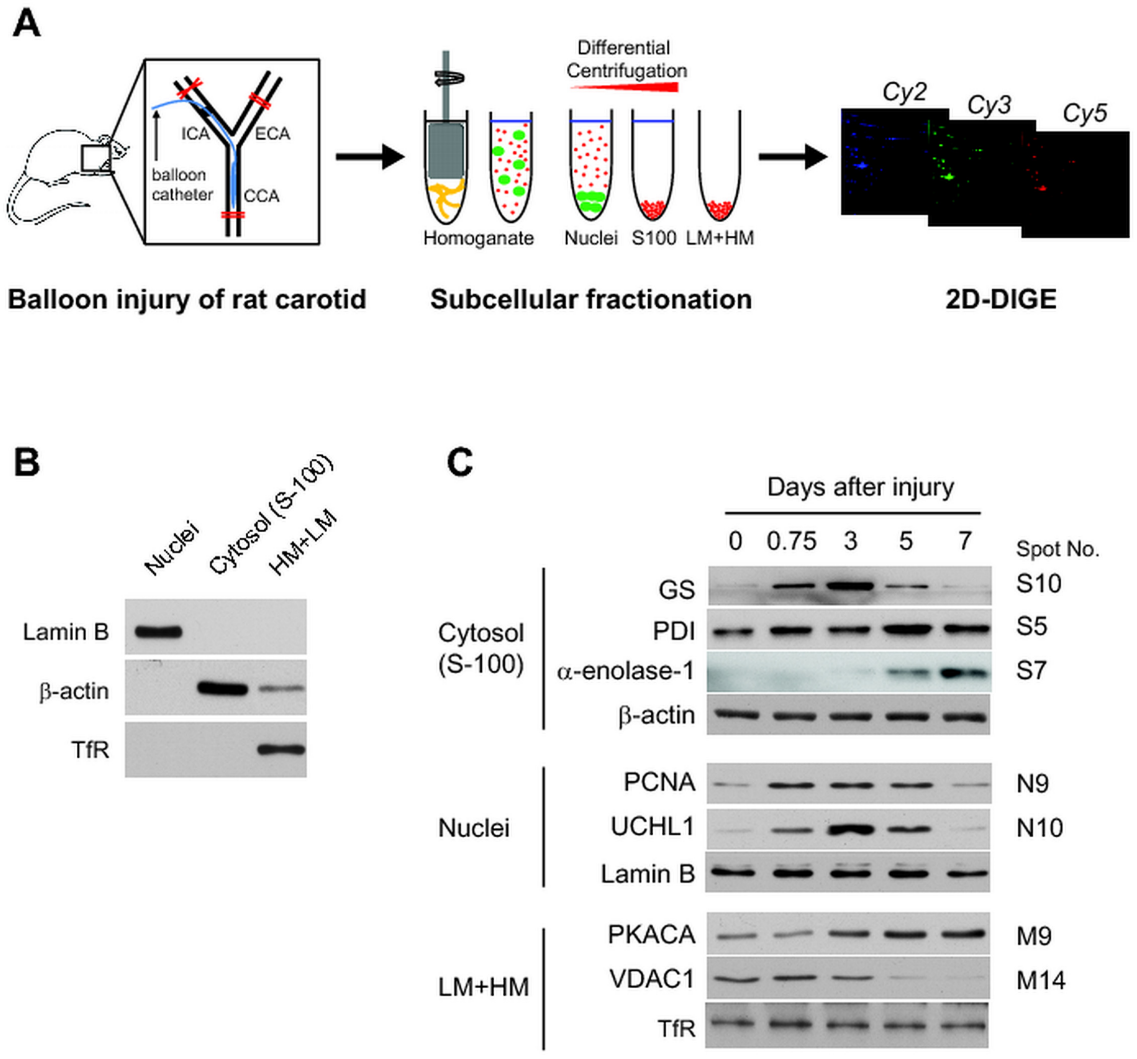
Analysis of proteome change in the injured arterial vessel. (A) Schematic presentation of quantitative proteome analyses. The rat carotid vessels were injured with a balloon catheter, as described in the Materials and Methods. The tissue homogenates of the injured rat carotids were fractionated into three subcellular fractions, labeled with fluorescent dyes (Cy2, Cy3, and Cy5), and separated on the 2D gels. ICA, internal carotid artery; ECA, external carotid artery; CCA, common carotid artery; LM, light membrane; HM, heavy membrane. (B) Quality control of the subcellular fractionations used in the 2D-DIGE analysis. The subcellular fractionation of the tissue homogenates was performed by differential centrifugation. Lamin B, β-actin, and transferrin receptor (TfR) were used as subcellular markers for nuclei, membranes, and cytosol (S-100), respectively. A representative immunoblot is shown (*n* = 3). (C) Western blot analysis of protein candidates selected from 2D-DIGE analyses. The differential expression of the indicated proteins during the recovery time after injury was evaluated by immunoblotting with specific antibodies. A representative blot is shown (*n* = 3). The corresponding spot numbers are indicated in parallel. GS, glutamine synthetase; PDI, protein disulfide isomerase; PCNA, proliferating cell nuclear antigen; UCHL1, ubiquitin carboxy-terminal hydrolase L1; PKACA, protein kinase A alpha catalytic subunit; VDAC1, voltage-dependent anion channel-1.

The physical injury of arterial vessels using a balloon embolectomy catheter causes the dysplastic neointimal thickening. This process involves the thrombosis-induced activation of VSMC hyperplasia following endothelial denudation. Experimentally, a balloon-induced injury of rat carotid artery is the best animal model that shows typical neointimal thickening within 10 days post injury. It is cost-effective and allows a reasonable amount of samples for histological and biochemical analyses because rat common carotid arteries are relatively long unbranched arterial vessels. It is significant that this model also represents the atherosclerotic plaque formation during which the VSMCs proliferates via a phenotypic change and form a fibrous cap in the plaque lesion [1]. As previously shown [26], the balloon injury induced a gradual increase of neointimal thickness in the lumen side of injured lesion (S1 Fig). Based on this kinetics, we chose the time points (0–7 days post injury) relevant to VSMC proliferation via a phenotypic change (contractile to synthetic) and then performed the two-dimensional differential gel electrophoresis (2D-DIGE) for quantitative proteome analysis (Fig 1A and S2A Fig). The tissue extracts were fractionated into three subcellular compartments (heavy/light membranes, nuclei, and cytosol) in order to maximize the number of proteins to be analyzed in 2D-DIGE (Fig 1B). As results, we analyzed more than 2,100 protein spots and found that 140 spots show a significant expression change. Among spots showing the differential expression the forty four proteins were identified by mass spectrometry (S1 Table and S2B–S2E Fig). It is noteworthy that most of the nuclei proteins identified (~80%) showed a transient change in protein level during recovery after balloon-induced injury; whereas, most proteins identified in membrane and cytosolic fractions showed a gradual increase or decrease. The differential expression of the identified proteins was again confirmed by immunoblotting with specific antibodies (Fig 1C), supporting that the proteome analyses were quantitative and accurate.

### Human VSMC-relevant candidate proteins are selected by in vitro cell assays

**Fig 2:**
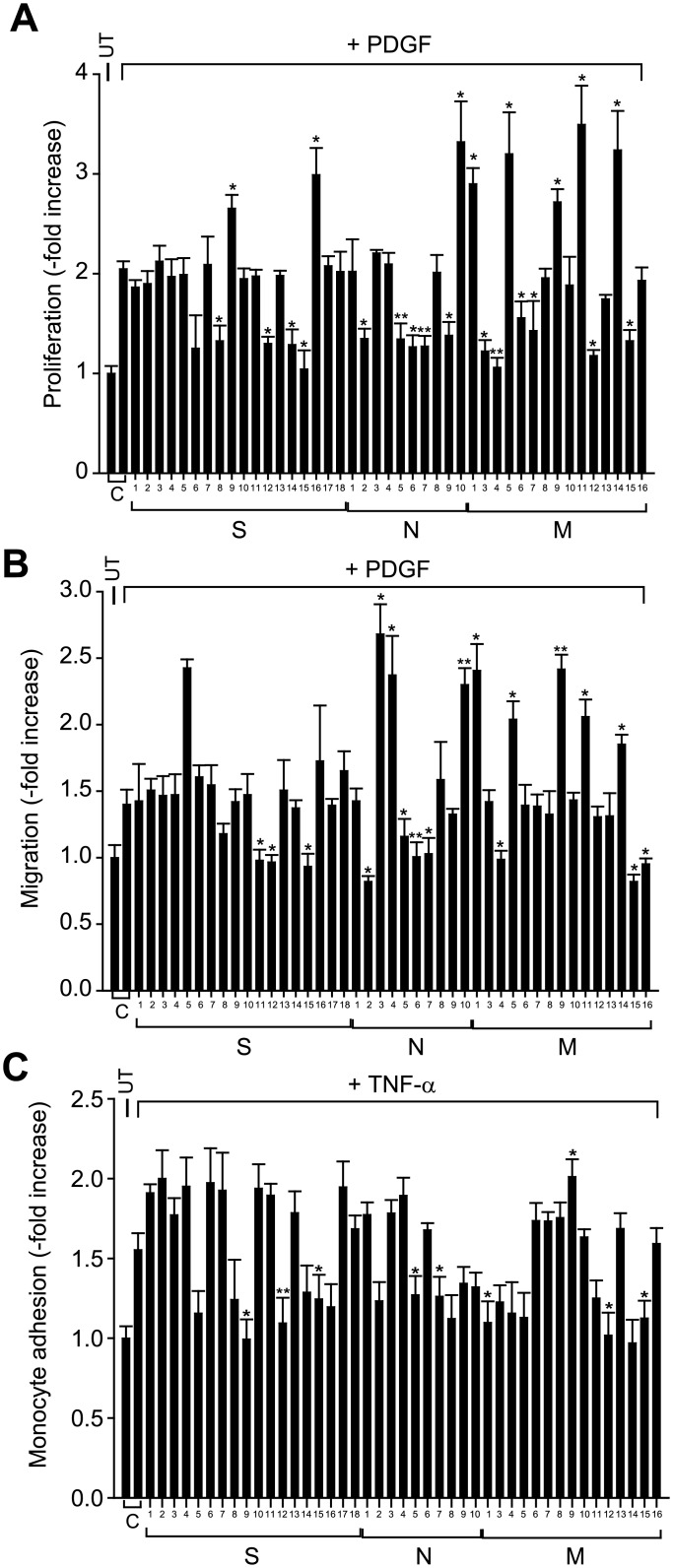
*In vitro* cell assays for functional validation of differentially-expressed protein candidates in HASMCs. HASMCs were transfected with the specific siRNA mixes listed in the S1 Table for 18 hr and stimulated with PDGF-BB (25 ng/ml) or TNF-α (10 ng/ml). Each experiment was performed in triplicate and the data in the graph are means ± S.D. of fold changes from three independent experiments (*P<0.02, **P<0.002). UT, untreated; C, control siRNA-transfected cells; S, cytosolic S-100; N, nuclei; M, membrane. The number designated each bar corresponds to the spot number.

A general concern has emerged whereby the expression pattern of a particular gene of interest can be different among the species. Thus, we decided to validate the cellular function of the rodent-origin 43 candidate proteins, except Ba1 (spot: M2), in human aortic VSMCs (HASMCs). The expression of each target gene was knocked down in the HASMCs by a reverse transfection with a mixture of four specific small interfering RNAs (siRNAs), as described in Materials and Methods (S2 Table). The high *in vitro* transfection efficiency was tested using a red fluorescent siGLO probe. The proliferation and chemotactic migration of aortic VSMCs were induced by PDGF-BB, which is a major VSMC growth factor; whereas, the monocyte adhesion to VSMCs was induced by tumor necrosis factor-α (TNF-α), a major pro-inflammatory cytokine. Both PDGF-BB and TNF-α are presumed to be produced mainly by macrophages in the atherosclerotic and balloon-injured lesions. From the extensive cell-based assays performed in triplicate, we found that depletion of 27 proteins showed positive and negative effects on one or more cellular activities; whereas, the depletion of residual 16 proteins had no effect on all three cellular activities (Fig 2 and Table 1). It supposed that the latter false positive proteins could be identified due possibly to the use of entire carotid vessel, including neointimal and medial layers, for proteomics analysis. Among those showing the effector activity, some of the candidate proteins were likely to be the master regulator in diverse cellular activities of human VSMCs. For example, the knockdown of PKAα catalytic subunit (PKACA) increased all three cellular activities; whereas, the knockdown of six proteins (Rab-15, Lactate dehydrogenase B, Intimal thickness-related receptor (ITR/GPR180), Oxidized LDL receptor (OLR1/LOX-1), and IL-12 receptor β2 (IL-12Rβ2)) significantly reduced three types of VSMC activities. It is noteworthy that the latter 5 positive effector proteins could be targeted for prevention of typical vascular SMC hyperplasia. Furthermore, we performed in-depth validation of two candidates, ITR and PKACA, as representative positive and negative effectors, respectively. RT-PCR showed the almost perfect knockdown of two gene expressions (S3A Fig). The *in vitro* cell assays confirmed that the depletion of ITR expression consistently reduced the PDGF-induced proliferation and chemotactic migration of human SMCs and inhibited the monocyte adhesion to human SMCs pre-activated by TNF-α treatment (S3B Fig). In contrast, the depletion of PKACA markedly augmented those SMC activities (S3C Fig). Collectively, the data indicate that the *in vitro* cell-based validation using human VSMCs effectively filtered out human-irrelevant false-positives among a number of candidate proteins screened from rodent disease model.

**Table 1:** List of proteins showing differential expression in the injured carotid artery and their cellular activities in VSMCs.

Spot	Gene	Protein	SwissProt	Loss-of-expression effect		
ID	ID	Name	Assession Number	Proliferation	Migration	Adhesion
**S1**	DNM1	Dynamin	O35303	-	-	-
**S2**	HSPCB	heat shock protein 90-beta	P34058	-	-	-
**S3**	AFP	alpha-fetoprotein	P02773	-	-	-
**S4**	CRMP1	Dihydropyrimidinase related protein	Q9JMG8	-	-	-
**S5**	GRP58	Protein disulfide-isomerase	P11598	-	-	-
**S6**	GC	Vitamin D-binding protein precursor	P04276	-	-	-
**S7**	ENO1	Enolase-1,alpha	P04764	-	-	-
**S10**	GLUL	Glutamine synthase	P09606	-	-	-
**S13**	ALDOC	Aldolase C	P09117	-	-	-
**S17**	NSF	N-ethylmaleimide sensitive factor	O88960	-	-	-
**S18**	LGALS1	Galectin-1	P11762	-	-	-
**N1**	NEO1	Unnamed protein product	P97603	-	-	-
**N8**	YWHAE	14-3-3 epsilon	P62260	-	-	-
**M8**	PRELP	PRELP	Q9EQP5	-	-	-
**M10**	ANXA5	Annexin A5	P14668	-	-	-
**M13**	STOM	stomatin	Q5XI04	-	-	-
**S12**	LDHB	Lactate dehydrogenase B	P42123	▽	▽	▽
**S15**	RAB15	Rab-15	P35289	▽	▽	▽
**N5**	OLR1	Oxidased low density lipoprotein receptor	O70156	▽	▽	▽
**N7**	ITR	Intima thickness-related receptor	Q5XIJ2	▽	▽	▽
**M15**	IL12RB2	IL-12 receptor beta 2	F1LRH7	▽	▽	▽
**N2**	LMNA	Lamin A	P48679	▽	▽	-
**N6**	PDHB	Pyruvate dehydrogenase beta	P49432	▽	▽	-
**M4**	PTPRE	Epsilon tyrosine phosphatase	B2GV87	▽	▽	-
**M12**	ANXA2	Calpatin 1 heavy chain(annexin A2)	Q07936	▽	-	▽
**S8**	SERPINA1	serine protease inhibitor 2c	P17475	▽	-	-
**S14**	RAB3D	Rab-16	Q63942	▽	-	-
**N9**	PCNA	PCNA	P04961	▽	-	-
**M3**	PLG	plasminogen	Q01177	▽	-	-
**M6**	CCT5	CCT(chaperonin containing TCP-1) epsilon subunit	Q68FQ0	▽	-	-
**M7**	AP2M1	Adaptor protein complex AP-2,mul	P84092	▽	-	-
**S11**	MDH1	Malate dehydrogenase-like enzyme	P04636	-	▽	-
**M16**	CYCS	Cytocrome C,somatic	P62898	-	▽	-
**M9**	PRKACA	PKA alpha catalytic subunit	P27791	▲	▲	▲
**N10**	UCHL1	UCH-L1	Q00981	▲	▲	-
**M5**	ANXA6	Annexin A6	P48037	▲	▲	-
**M11**	ANXA1	Lipocortin 1 (Annexin A1)	P07150	▲	▲	-
**M14**	VDAC1	VDAC-1	Q9Z2L0	▲	▲	-
**S16**	HSPB1	heat shock 27	P42930	▲	-	-
**N3**	GTF2F1	general transcription factor IIF polypeptide 1	Q6AY96	-	▲	-
**N4**	SERPINF1	serine protease inhibitor,clade F	Q80ZA3	-	▲	-
**S9**	PLD2	Phospholipase D2	F1LQD7	▲	-	▽
**M1**	MBC2	membrane bound C2 domain containing protein	Q9Z1X1	▲	▲	▽

▲ and ▽ symbols indicate the significant increase and decrease of each cell activity, respectively (filtered by *P* value below 0.02). - symbol indicates the unchanged cell activity.

### Protein interaction network elicits novel proteins involved in the SMC hyperplasia

**Fig 3:**
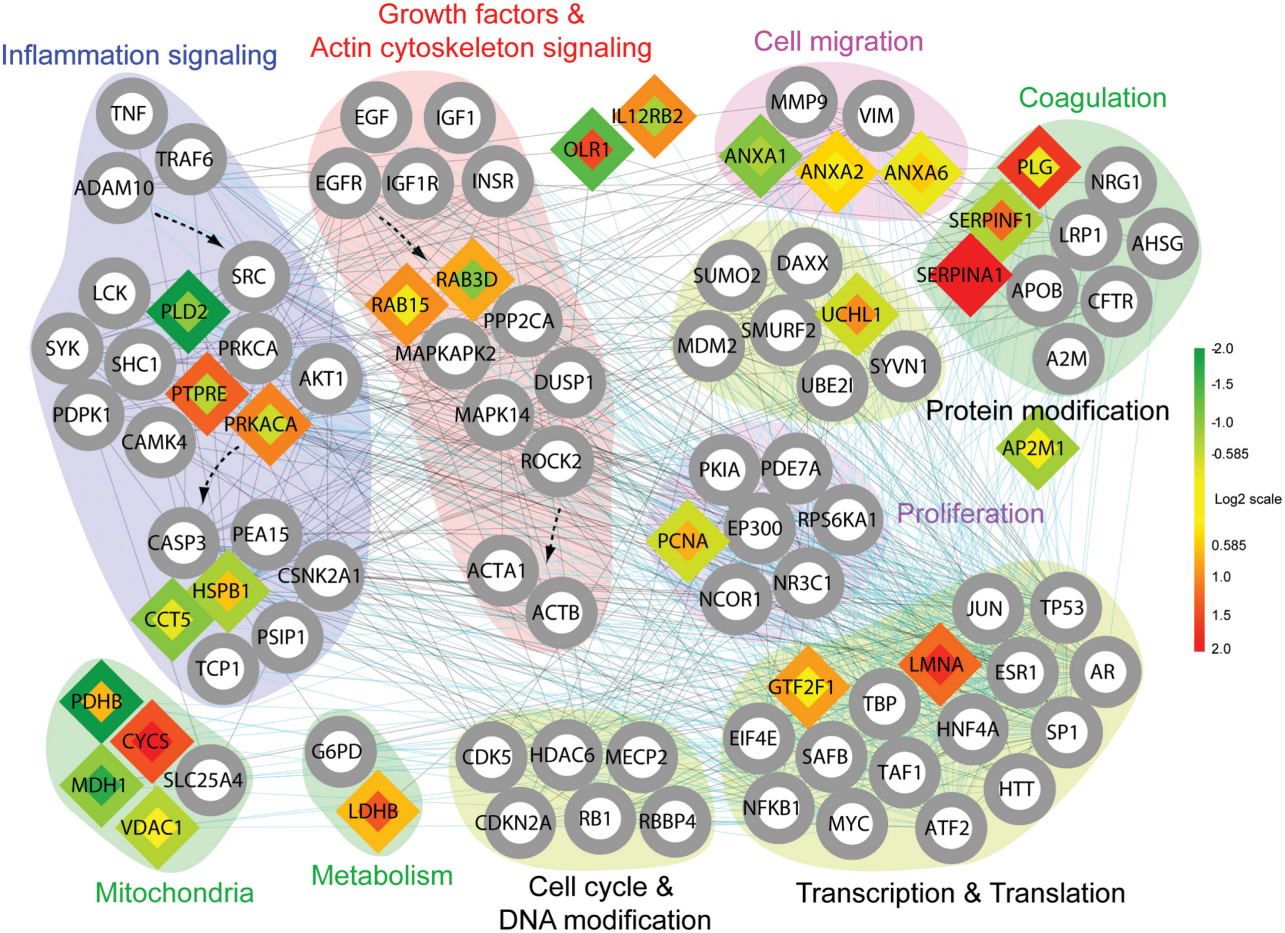
A network model of proteins related to the VSMC hyperplasia in vascular injury response. The nodes with the same GOBPs or KEGG pathways were grouped into the same modules, each of which was named by the corresponding GOBP or KEGG pathway. The 25 proteins are denoted by the diamond nodes. Inner and outer node colors represent the changes in abundances of the 25 proteins at 18 hr and 7 days after balloon-induced injury, respectively. Color bar, gradient of log2-fold-changes. Red and green colors represent up- and down-regulation of the proteins, whereas yellow represents no changes in abundance. White circular nodes with gray boundaries denote the proteins added to connect the 25 proteins. Black and blue edges represent protein-protein and protein-DNA interactions, respectively.

To understand a functional correlation among 27 selected proteins, we then constructed a network model showing the map of the protein-protein or protein-DNA interactions (Fig 3). ITR and membrane-bound C2 domain-containing protein were omitted due to the lack of the interaction data and significance in gene ontology biological processes (GOBPs). The network model showed that the 23 proteins entirely participated in growth factor receptor tyrosine kinase and actin cytoskeleton signaling pathways, as well as cellular responses associated with these signaling pathways, such as cell migration, coagulation, and proliferation-related processes (transcription/translation, protein modification, and metabolism). Thus, the network model supports that our proteome analyses indeed identified the key signaling molecules essential for the VSMC proliferation and migration. In addition, we found that the two receptor proteins, OLR1 and IL-12Rβ2, do not belong to any of the signaling clusters in the network.

**Fig 4:**
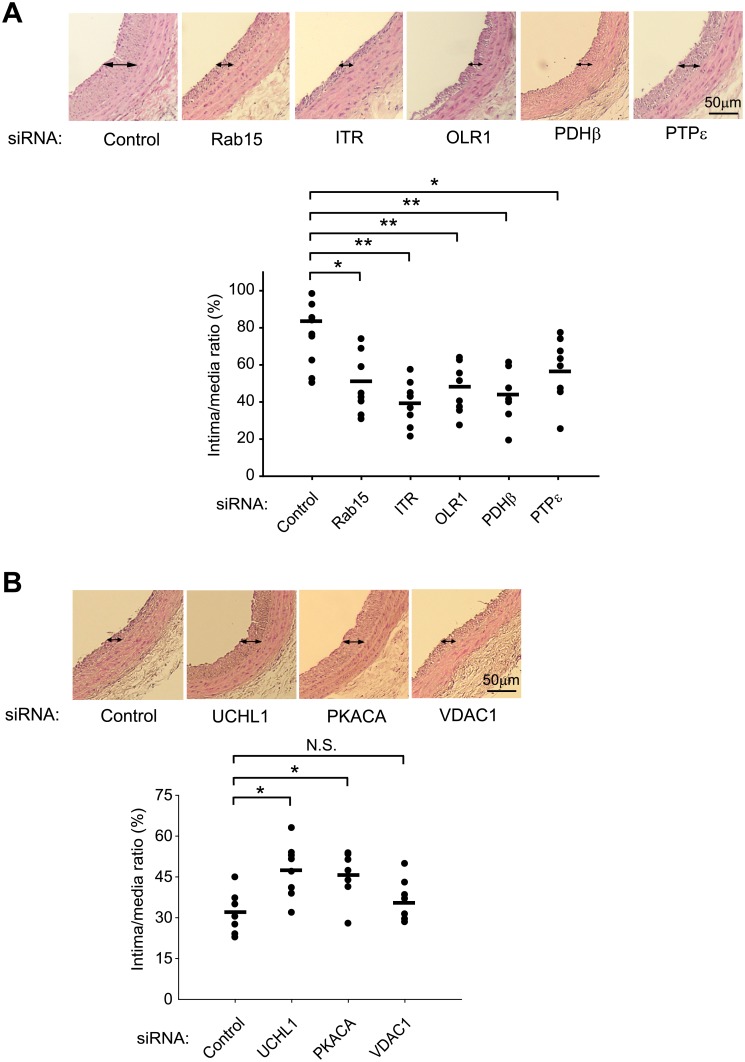
*In vivo* functional effect of the depletion of protein candidates on neointimal hyperplasia. The rat carotid arteries were injured and transfected by a catheter-mediated delivery of the indicated siRNAs as described in the Materials and Methods. After a 10-day recovery, the carotid tissue sections were prepared and subjected to the histological analyses. Representative HE-stained images are shown. Data in the graph are means ± S.E.M. of intima versus media ratio measured from HE-stained carotid samples (*n* = 7 rats per group, *P<0.05, **P<0.01).

Based on the network analysis, we performed an *in vivo* validation of novel node proteins in the pathway clusters using a balloon-induced injury model of rat carotid. To do it, we knocked down the protein expression by an *in vivo* transfection of specific siRNAs to the injured lesion as described in Methods and Materials. The efficacy of the *in vivo* transfection was tested using a fluorescent dye-conjugated siRNA (siGLO), suggesting that the siRNA transfection into the medial SMC layer can be achieved only after a balloon-induced injury (S4A Fig). The *in vivo* transfection was further validated by targeting a SMC growth factor receptor PDGFRβ. The siRNA-mediated knockdown of PDGFRβ in the injured carotid vessels was achieved (S4B and S4C Fig) and it consequently reduced the neointimal thickening (S4D Fig). Subsequently, we performed the siRNA-mediated knockdown of the key node proteins (S4E Fig). The knockdown of each of six proteins (Rab-15, ITR, OLR1, PDHβ, IL-12Rβ2, and PTPε) that had shown the positive effects on SMC proliferation and migration indeed elicited a significant reduction in the balloon-induced neointimal thickening (Fig 4A). In contrast, the knockdown of two proteins (UCH-L1 and PKACA) that had shown the negative effect on the SMC activities augmented the neointimal thickening (Fig 4B). The knockdown of VDAC1 had no effect on the neointimal thickening, which may represent a discrepancy between rodent and human systems.

**Fig 5:**
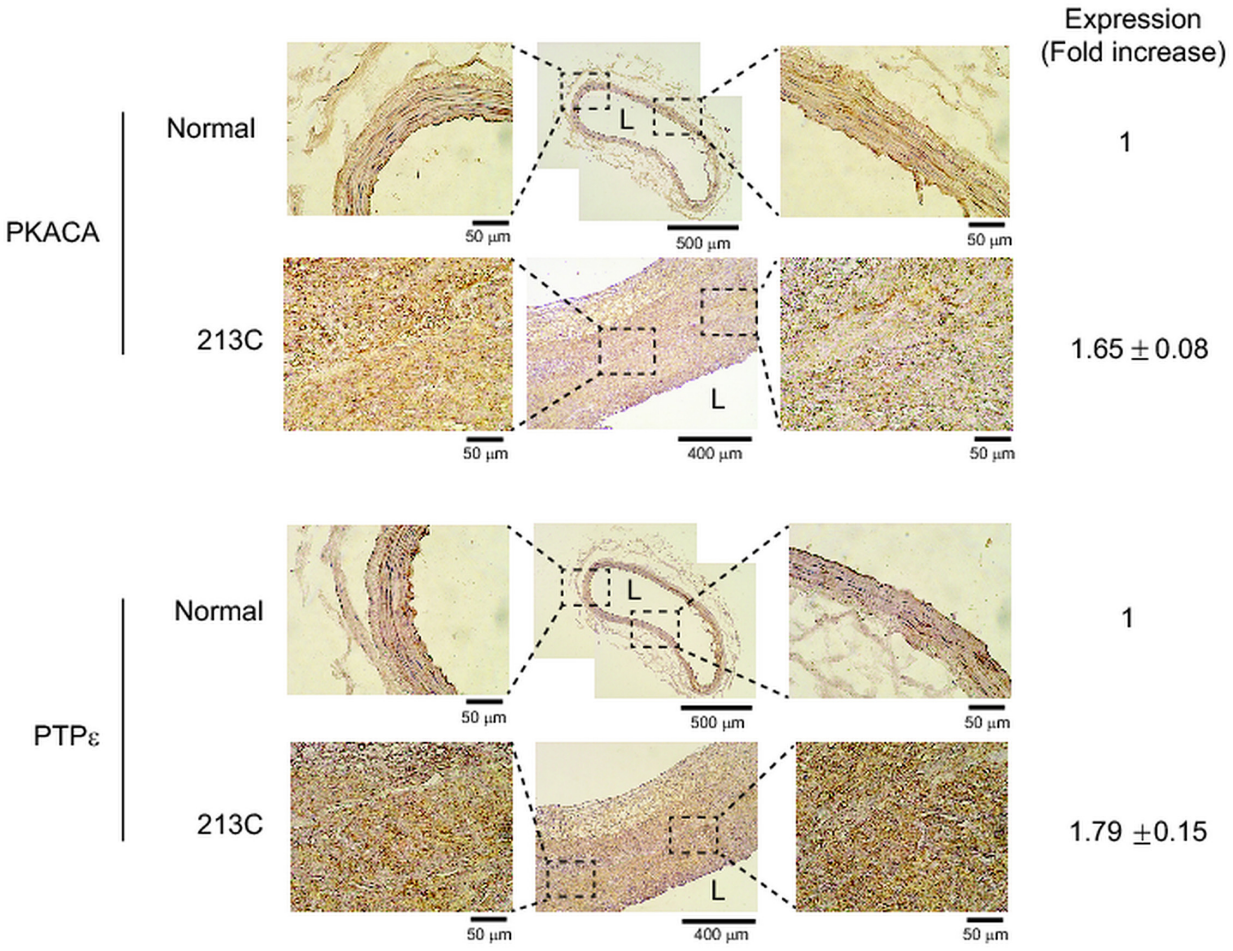
Increased expression of PKACA and PTPε in human carotid arterial sections with atherosclerotic lesions. Immunohistochemistry was performed using the paraffin-embedded tissue sections of human carotid arteries with thickened intimal lesions (Origin Technologies, Rockville, MD, USA). The indicated proteins were stained with specific immunohistochemistry-compatible antibodies. Frozen sections of normal peripheral arteries were stained for control. The 3’,3’-diaminobenzidine (DAB)-stained images are obtained from 5 patient carotid tissue sections and quantified with the HistoFAXS Tissue Analysis System (TissueGnostics, USA). The expression level is shown as a fold increase of DAB intensities in patient samples versus that in normal vessel. A representative image with patient identification number is shown. The letter “L” indicates the vascular lumen.

To elicit a relevance to human vascular disease, we performed immunohistochemistry (IHC) using carotid arterial sections of human patients with atherosclerotic lesion (Fig 5 and S5 Fig). The levels of two of the validated proteins (PKACA and PTPε) were increased in the atherosclerotic lesions by 1.7- and 1.8-fold than those of normal vessel, respectively. This result confirms that our proteomics screening using a balloon-injured rat model elicited the human-relevant atherosclerosis target proteins.

### OLR1 is a multifaceted signal regulator in human SMCs

**Fig 6:**
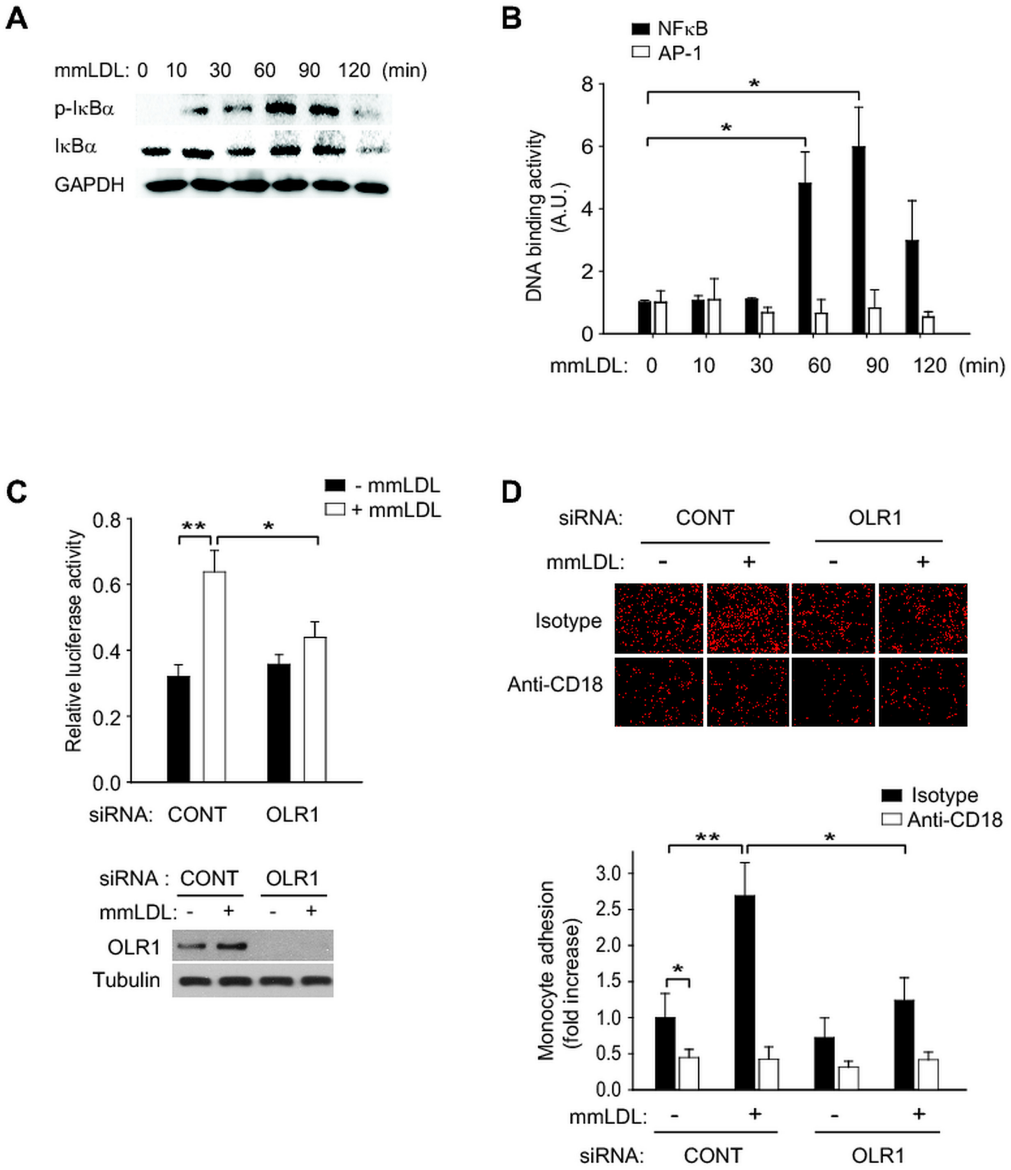
OLR1 is the oxidized LDL receptor in VSMCs. (A) OxLDL-induced IκB phosphorylation and degradation in VSMCs. The HASMCs were stimulated with OxLDL (100 μg/ml) for the indicated time periods. (B) OxLDL-induced DNA-binding activity of NF-κB p65 and AP-1 in VSMCs. The DNA-binding activity was measured using ELISA-based colorimetric TransAM kit (Active Motif, Carlsbad, CA), a 96-well plate with immobilized oligonucleotides encoding NF-κB or AP-1 consensus binding sequences. Each assay was performed in triplicate and the data in the graph are means ± S.D. of the absorbance units (*A*_450_) from three independent experiments (*P<0.005). (C) OxLDL-induced NF-κB transcriptional activity in VSMCs transfected with control or OLR1 siRNA. HASMCs were transfected with a NF-κB-dependent luciferase reporter vector followed by siRNA transfection. Cells were incubated in the absence or presence of OxLDL for 6 hr. The relative luciferase activity was normalized to the β-galactosidase activity and the data in the graph are means ± S.D. of the luciferase activities from three independent experiments (*P<0.02, **P<0.002). (D) OxLDL-induced monocyte adhesion in VSMCs transfected with control or OLR1 siRNA. After the siRNA transfection, the HASMCs were stimulated with OxLDL for 24 hr and then preincubated in the presence of isotype control or anti-CD18 neutralizing antibody before the cell adhesion assay. Representative images are shown and the data in the graph are means ± S.D. of fold change relative to untreated control cells from three independent experiments (*P<0.01, **P<0.005).

We were then particularly interested in the OLR1 known as an endothelial receptor. The function of OLR1 in VSMCs was unknown although its expression was reported in VSMCs [27]. We first tested whether the OLR1 functions as an oxidized LDL receptor in VSMCs. Treatment with minimally oxidized LDL (mmLDL) induced the serine phosphorylation-dependent degradation of IκB in VSMCs (Fig 6A) and subsequently enhanced the DNA-binding activity of NF-κB (Fig 6B). The mmLDL-induced NF-κB activation was completely blocked by the OLR1 knockdown (Fig 6C). These results indicate that VSMCs contain OLR1 as an endogenous oxidized LDL receptor that mediates the NF-κB activation. More importantly, the OLR1 knockdown inhibited the monocyte adhesion to the VSMCs activated by mmLDLs, which was completely blocked by a neutralizing antibody against intercellular adhesion molecule-1 (Fig 6D). Considering that the ICAM-dependent monocyte adhesion is crucial for the initiation of inflammatory response, it is likely that the OLR1 induction in VSMCs by vascular injury may contribute to the inflammation, thereby exacerbating VSMC hyperplasia.

**Fig 7:**
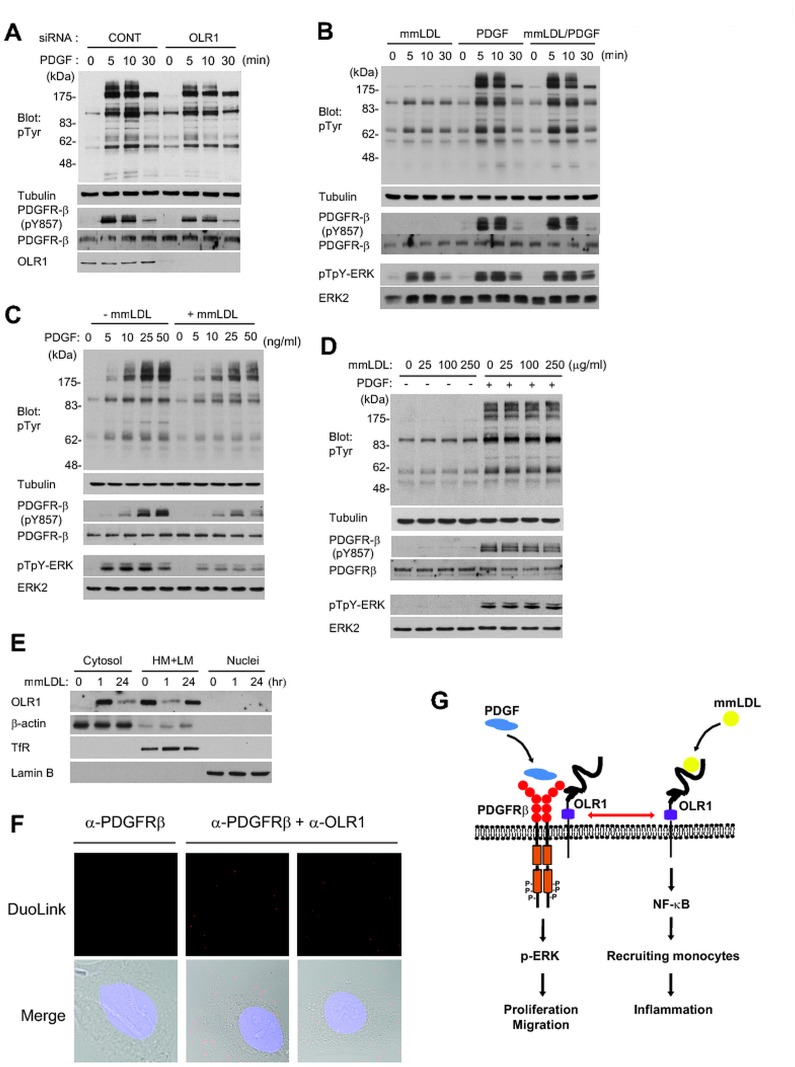
OLR1 is associated with the PDGF signaling in VSMCs. (A) PDGF-dependent tyrosine phosphorylation in VSMCs transfected with control or OLR1 siRNA. The HASMCs were starved in serum-free culture media for 18 hr and the stimulated with PDGF-BB (25 ng/ml) for the indicated times. The protein tyrosine phosphorylation was detected using anti-phosphotyrosine antibody (4G10) and the PDGFR activation was measured by immunoblotting the Tyr857 phosphorylation on PDGFRβ. A representative immunoblot is shown (*n* = 3). (B—D) Effect of OxLDL treatment on PDGF-induced tyrosine phosphorylation in VSMCs. The serum-starved VSMCs were co-treated with PDGF-BB and OxLDL (B) or pretreated with OxLDL for 1 hr (C) and 24 hr (D) before PDGF-BB treatment. The activation of MAP kinase ERK was measured by phospho-specific antibody (pTpY). A representative immunoblot is shown (*n* = 3). (E) Subcellular distribution of OLR1 in OxLDL-treated VSMCs. Cells were treated with OxLDL (100 μg/ml) for 1 hr or 24 hr and the cell extracts were then separated to the subcellular fractions by differential centrifugation. A representative immunoblot is shown (*n* = 3). (F) The proximity ligation assay showing the OLR1 and PDGFRβ interaction in the plasma membrane of VSMCs. The proximity ligation assay was performed using DuoLink in situ probes with the indicated antibodies. DIC and DAPI-stained images are merged with DuoLink fluorescence image (merge). Representative images from two experiments are shown. (G) OLR1 plays a dual receptor function in VSMCs. OLR1 is expressed in VSMCs and also induced in the arterial vessels by an endothelial denudation. OLR1 primarily functions as an oxidized LDL receptor in VSMCs that mediates the NF-κB-dependent inflammatory response. OLR1 itself enhances the PDGF-induced signal transduction including PDGFRβ and ERK phosphorylation. Therefore, the OLR1 is the first SMC receptor that mediates the pro-inflammatory and proliferative responses in a ligand-dependent and -independent manner, respectively. Purple colored box is a leucine repeat in OLR1.

Since OLR1 played a role in PDGF-induced proliferation and migration, we further examined the effect of the OLR1 on PDGF signaling. The knockdown of OLR1 expression decreased the protein tyrosine phosphorylation including PDGFRβ phosphorylation in response to PDGF stimulation (Fig 7A). We then hypothesized that OLR1 would function as a co-receptor for the full activation of PDGFRβ in VSMCs. To test this hypothesis, we explored whether there is a functional interaction between PDGFRβ and OLR1 in VSMCs. The mmLDL treatment neither induced the PDGFRβ phosphorylation in VSMCs nor interfered with the PDGF-induced tyrosine phosphorylation (Fig 7B). This result indicates that the mmLDL-OLR1 interaction was PDGFRβ-independent. In addition, a slight activation of tyrosine phosphorylation and ERK kinase by mmLDL alone might be due partly to the formation of hydroxynonenal-PDGFR adduct [28]. However, the 1-hr pretreatment of mmLDL significantly reduced the PDGF-induced tyrosine phosphorylation (Fig 7C). In contrast, the 24-hr pretreatment of mmLDL at various doses had no effect on the PDGF signaling (Fig 7D). It is known that the scavenger receptors like OLR1 are endocytosed after the ligand engagement [29, 30]. Indeed, the OLR1 was endocytosed from membrane to cytosolic fraction by 1-hr treatment of mmLDLs, whereas it was completely recovered in the membrane after 24 hr (Fig 7E). To further identify the interaction between OLR1 and PDGFRβ, we performed the proximity ligation assay using DuoLink. The combination of anti-PDGFRβ and anti-OLR1 antibodies, not the anti-PDGFRβ antibody alone, showed that OLR1 interacted with PDGFRβ in close proximity in the plasma membrane of VSMCs (Fig 7F). Together, these results suggest that the mmLDL-dependent clearance or genetic depletion of OLR1 from the plasma membrane may dampen the PDGF-PDGFRβ signaling events in VSMCs.

## Discussion

Numerous proteomic and transcriptomic analyses have been carried out in the past decade to identify biomarkers and target genes related to atherosclerosis. The direct tissue proteomics using human coronary atherosclerotic plaques have identified several tens of proteins involved in the plaques, several of which were validated by immunohistochemistry [31]. The CardioGene study of human patients with in-stent restenosis was designed by the National Heart, Lung and Blood Institute, NIH (USA) and included proteomics of accessible plasma samples, not the stent-implanted aortic tissue samples [32]. The rodent models, such as ApoE-deficient mice fed with high-fat diet or a surgically-induced injury of rat carotid artery, were subjected to the proteomics and revealed some biomarker candidates and expression profile [33, 34]. However, we realized that most of these studies have not performed in-depth validation processes. Therefore, we focused on the multi-layer validation after the quantitative proteomics screening: 1) Use of rodent model for simplicity and reproducibility, 2) *In vitro* validation using human aortic VSMCs for human relevance, and 3) Evaluation of functional correlation of the protein candidates by informatics. Such proteomics and bioinformatics processes produced a list of candidate proteins that show little overlap with the previous proteomic studies. In particular, we could select the valuable candidates (e.g. OLR1, PDHβ, and UCHL1) among proteins, whose expression has been restored to control levels by day 7 after the balloon injury, by the subsequent *in vitro* cell assays. Considering that such proteins with a transient expression change are usually excluded from proteomics study, our study emphasizes the importance of multi-layer validation steps following the tissue proteomics. Furthermore, some proteins strongly support the significance of our vascular proteomics work in relation to vascular thickening. For example, ITR was shown to be a G protein-coupled receptor inducing an experimental intimal thickening [35]. PLD2 was indirectly involved in the angiotensin II-induced neointimal thickening in the balloon-injured artery [36]. Takami *et al* have shown that UCH-L1 was highly expressed in the neointima of the balloon-injured carotid artery and was also detected in atherosclerotic lesions from human carotid arteries [37]. Since most candidate proteins have not been described in vascular injury or atherosclerosis, we constructed a network model showing functional links between the candidates. The interaction network model revealed that 23 out of 27 proteins are associated with the growth factor receptor-mediated actin cytoskeleton reorganization signaling and its related processes: cell migration, coagulation, and proliferation. The network model also offers the insulin-like growth factor-1 (IGF-1) as one of the most significant extracellular stimuli. In fact, the IGF-1 expression in VSMCs was shown to associate with insulin insensitivity [38]. The IGF-1 receptor was detected in atherosclerotic plaques together with IL-12 and IFNγ [8].

In addition, we studied a novel cellular function of OLR1 in VSMCs because it does not belong to the growth factor signaling clusters in our network model. Like other scavenger receptors implicated in the atherosclerosis [5, 16], the OLR1 mediated the OxLDL-induced NF-κB activation in VSMCs, which in turn augmented the ICAM-dependent monocyte adhesion onto the activated VSMCs. More striking finding is that the depletion or internalization of the OLR1 impaired the activation of PDGFRβ, a major growth factor receptor of VSMCs. This evidence suggests a novel function of OLR1 as a potential co-receptor for PDGFRβ, similar to a co-receptor neuropilin-1 for vascular endothelial growth factor receptor-2 [39]. Thus, it is likely that OLR1 is the first scavenger receptor that simultaneously coordinates the growth signaling and the inflammatory response in VSMCs (Fig 7G). If this is the case, it is suspected that a PDGFR-targeted therapy against atherosclerosis and in-stent restenosis may cause a medical complication in hypercholesterolemia or hyperlipidemia patients [40–43].

In summary, our proteomic screening reveals many potential targets, particularly OLR1, for vascular diseases with the SMC hyperproliferation.

## Supporting Information

S1 Fig Histology of common carotid arteries after balloon-induced injury in rats. Neointimal thickening was examined during recovery time in balloon-injured carotid artery. Representative HE-stained images for normal vessels, 3-day, 7-day, and 14-day post injury are shown. Arrow indicates the thickened neointimal layer. Data in the graph are means ± SEM of intima versus media ratio measured from HE-stained carotid samples (*n* = 4 rats per group).
(PDF)

S2 Fig 2D-DIGE and mass spectrometric analyses of protein spots with differential expression (Related to Fig 1). (A) Typical 2D-DIGE fluorescence images for S-100 cytosolic fraction. The protein samples from two consecutive time points were differently labeled with Cy3/Cy5 dyes. The internal standard (Std) was labeled with Cy2 dye. (B—D) Quantitative analyses of the differentially-expressed protein spots on 2D-DIGE gels. The Cy2/Cy3/Cy5 fluorescences were quantified using the DyCyer software tools in a Typhoon 9400 imager. The amount of Cy3/Cy5 fluorescence was normalized by that of Cy2 fluorescence and plotted in the histograms. Data in the histogram show the mean ± S.D. of the fold increase of the fluorescence intensities versus zero time point (*n* = 3 repeated experiments, *P<0.01, **P<0.001). The representative silver-stained 2D gels for cytosolic (B), nuclei (C), and membrane (D) fractions are shown. The position of selected protein spots with differential expression are indicated by arrows with serial spot numbers. The pH gradients and molecular weights are indicated on the horizontal and vertical axes, respectively. The graphs show. (E) LC-MS/MS spectra. The MS/MS spectrum of [M + H]^+^ ions of one of the peptides derived from the indicated protein is shown. PTPRE, Epsilon tyrosine phosphatase; ANXA2, Annexin A2; F1LRH7, IL-12 receptor beta 2; HS90B, Hsp90β; A1AT, Serine protease inhibitor 2c; OLR1, Oxidized LDL receptor-1; G3V799, Intima thickness-related receptor; 1433E, 14-3-3 epsilon; RAB15, Rab-15.
(PDF)

S3 Fig Reciprocal regulation of SMC activities by ITR and PKACA. (A) Expression of ITR and PKACA mRNAs in HASMCs transfected with control or specific siRNAs. Total RNA was extracted from the HASMC cells using Trizol reagent (Invitrogen) and subjected to a reverse transcriptase (RT) reaction using ImProm-II reverse transcription system (Promega). The resulting cDNAs was amplified by PCR using Taq DNA polymerase (Promega) and the PCR products were visualized by agarose gel electrophoresis and ethidium bromide staining (BIPS system, Bio-Rad). (B) Negative effect of the ITR knockdown on PDGF-dependent SMC proliferation/migration and TNF-α-dependent monocyte adhesion. (C) Positive effect of the PKACA knockdown on PDGF-dependent SMC proliferation/migration and TNF-α-dependent monocyte adhesion. Each experiment was performed in triplicate and the data in the graph are means ± S.D. of fold increases from three independent experiments (*P<0.01, **P<0.005).
(PDF)

S4 Fig Catheter-mediated intramural delivery of siRNA-transfection carrier premixes to the balloon-injured carotid arteries (Related to Fig 4). (A) *In vivo* siRNA transfection test. The fluorescent scrambled siRNA called siGLO (Dharmacon) was introduced into the lumen of normal or balloon-injured rat common carotid arteries with or without siPORT NeoFX transfection reagent. Note that the siGLO are transfected to the neointimal cells only when mixed with transfection reagent in the balloon-injured arteries. Arrow indicates the thickened neointimal layer. Representative HE-stained and fluorescence images from two independent experiments are shown. (B and C) *In vivo* transfection of rat PDGFRβ siRNAs sufficiently reduces the PDGFRβ expression in rat carotid arteries. The level of PDGFRβ was shown by immunoblotting (B) and immunofluorescence staining (C). (D) Neointimal thickening in the balloon-injured carotid artery is reduced by the PDGFRβ knockdown. Representative HE-stained images are shown. Data in the graph are means ± SEM of intima versus media ratio measured from HE-stained carotid samples (*n* = 3 rats per group). (E) Reduction of the target gene expression in the balloon-injured carotid arteries after the *in vivo* transfection of specific siRNAs. Total RNA from carotid vessels was purified using the RNeasy fibrous tissue kit (Qiagen). RNA (2 μg) was reverse transcribed using ImProm-II RT system (Promega). The real-time PCR was performed using specific primers in the presence of SYBR Green (Applied Biosystems) inside a fluorescent temperature cycler (ABI Prism 7000 sequence detection system, Applied Biosystems). The fluorescence signals were quantified by a comparative cycle threshold method. After finishing these cycles, a melting point was checked for specificity. The β-actin mRNA and 18S rRNA were used as endogenous reference genes. The expression level in the graph is means ± S.D. of fold changes relative to control-transfected samples (*n* = 3 rats per group).
(PDF)

S5 Fig Increased expression of PKACA and PTPε in carotid lesions of human patients with vascular narrowing (Related to Fig 5). Immunohistochemistry was performed using the paraffin-embedded tissue sections of human carotid arteries with thickened intimal lesions (Origin Technologies, Rockville, MD, USA). The indicated proteins were stained with specific immunohistochemistry-compatible antibodies. The 3’,3’-diaminobenzidine (DAB)-stained images of carotid tissue sections are labeled with patient identification numbers.
(PDF)

S1 Table Summary of mass spectrometric analyses for vascular proteomes with differential expression. (PDF)

S2 Table List of siGENOME siRNAs used for *in vitro* cell assays. (PDF)
