# Supplementary figures and images for: Vascular Proteomics Reveal Novel Proteins Involved in SMC Phenotypic Change: OLR1 as a SMC Receptor Regulating Proliferation and Inflammatory Response
Source: PLoS One. 2015 Aug 25;10(8):e0133845. doi: 10.1371/journal.pone.0133845 (PMC4548952; doi:10.1371/journal.pone.0133845)

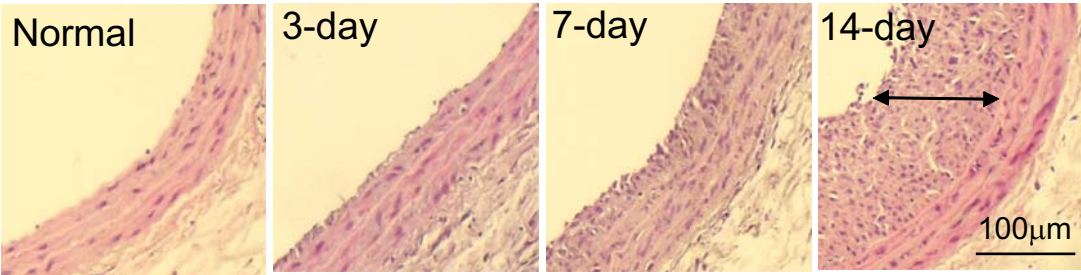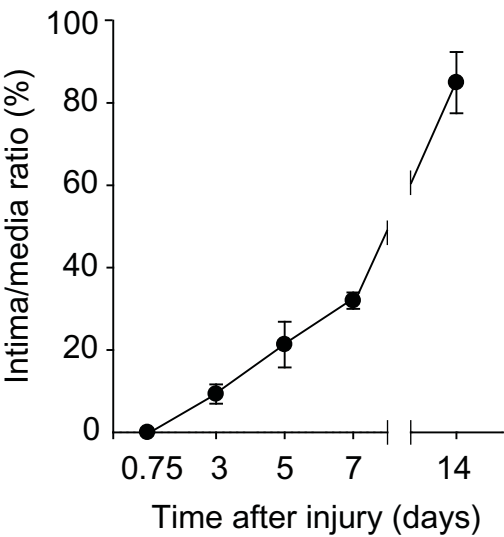

Supplement: S1 Fig — Neointimal thickening was examined during recovery time in balloon-injured carotid artery. Representative HE-stained images for normal vessels, 3-day, 7-day, and 14-day post injury are shown. Arrow indicates the thickened neointimal layer. Data in the graph are means ± SEM of intima versus media ratio measured from HE-stained carotid samples (n = 4 rats per group). (PDF) [file pone.0133845.s001.pdf]

A

DIGE-S100

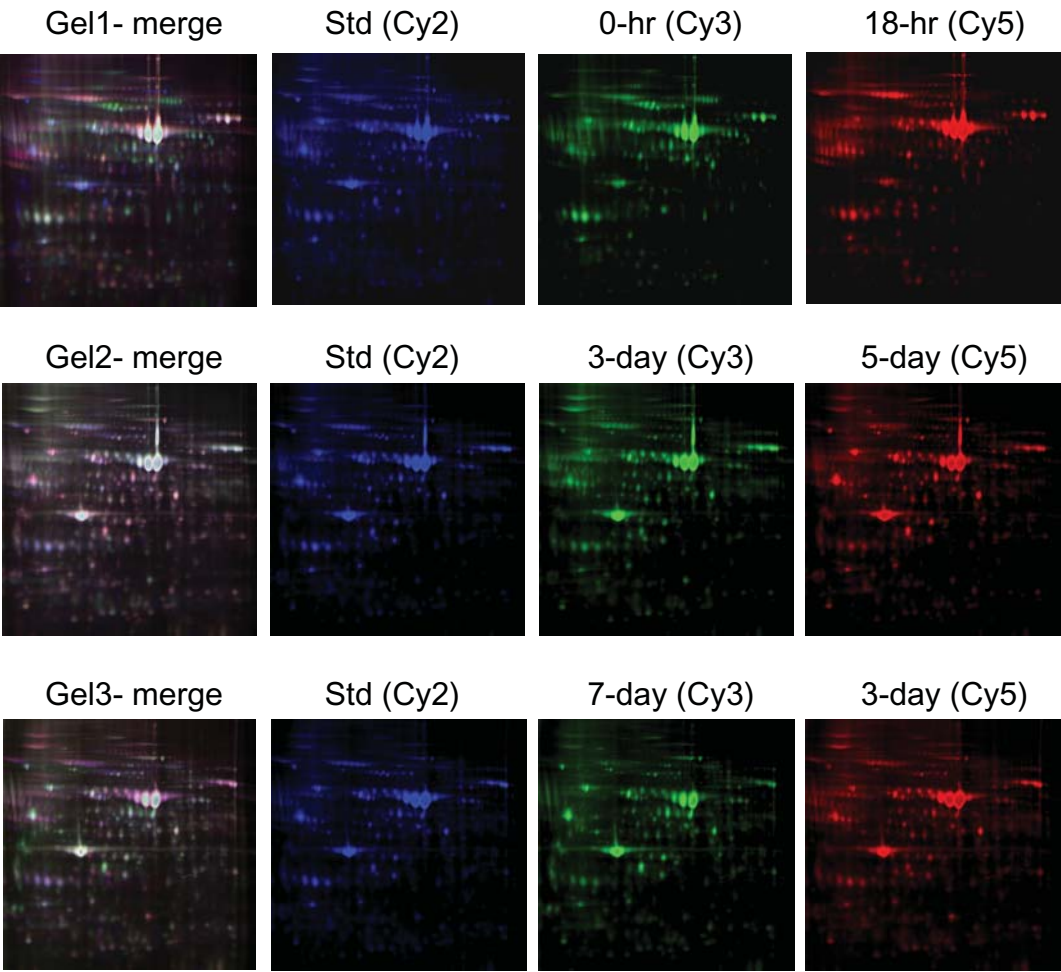

B

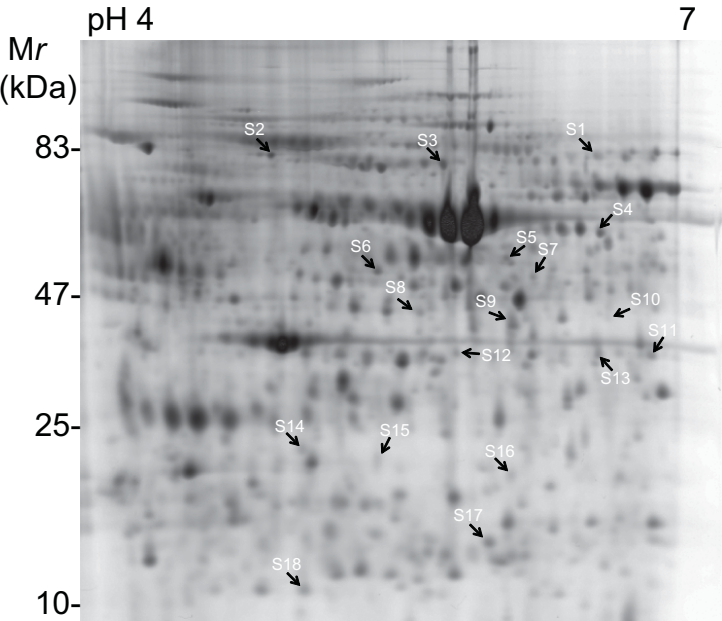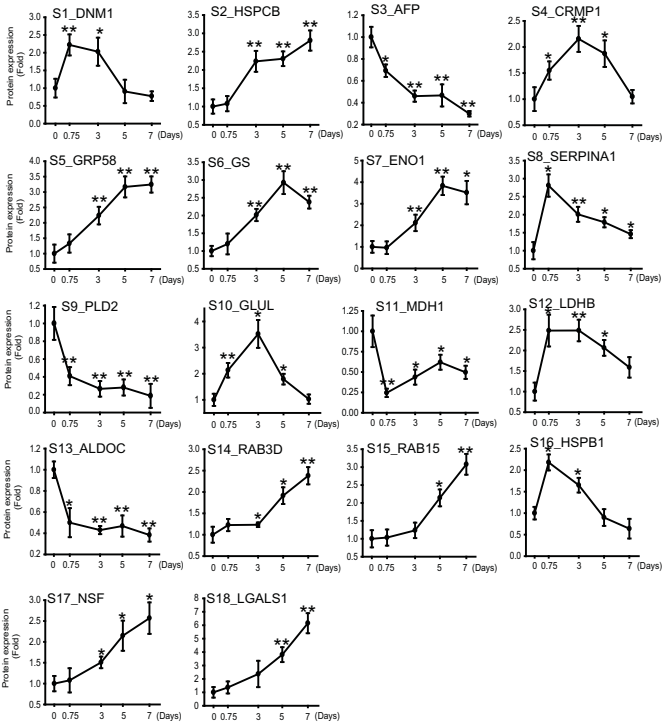

C

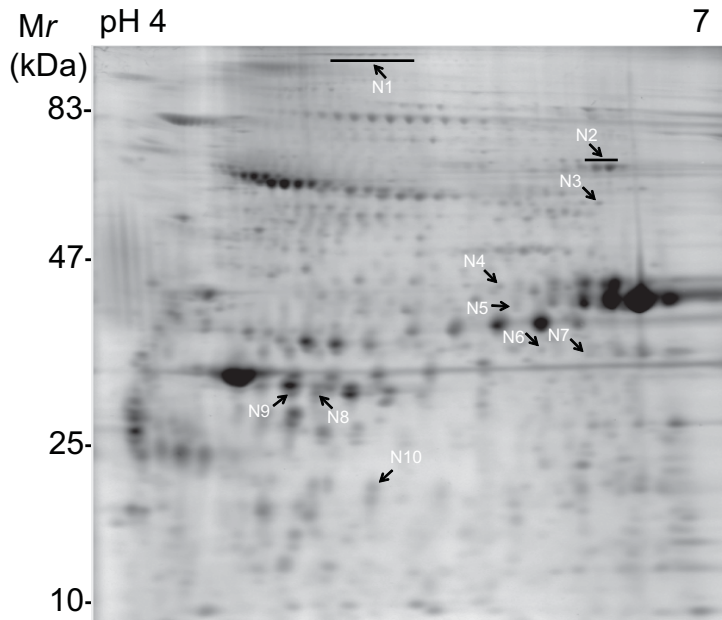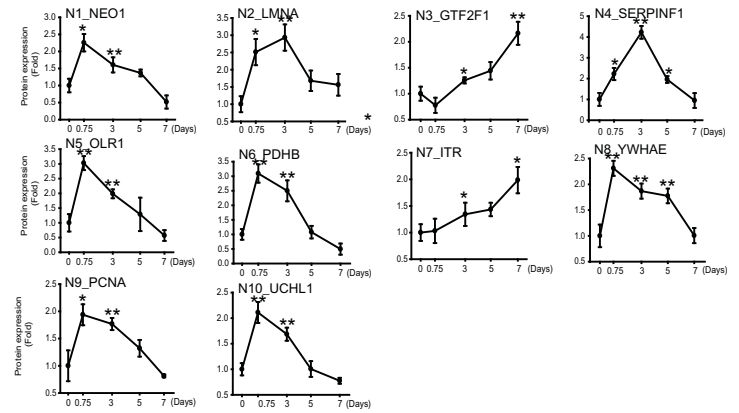

D

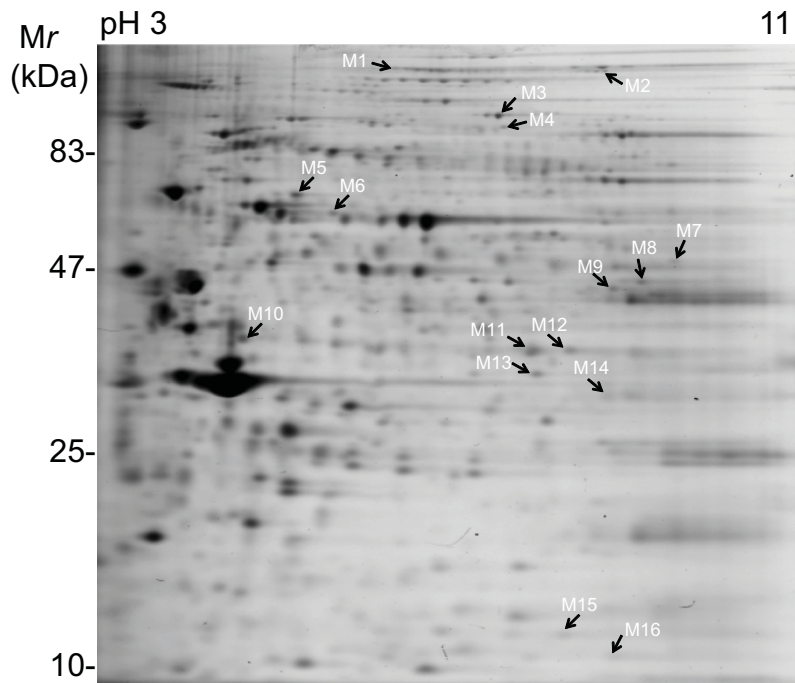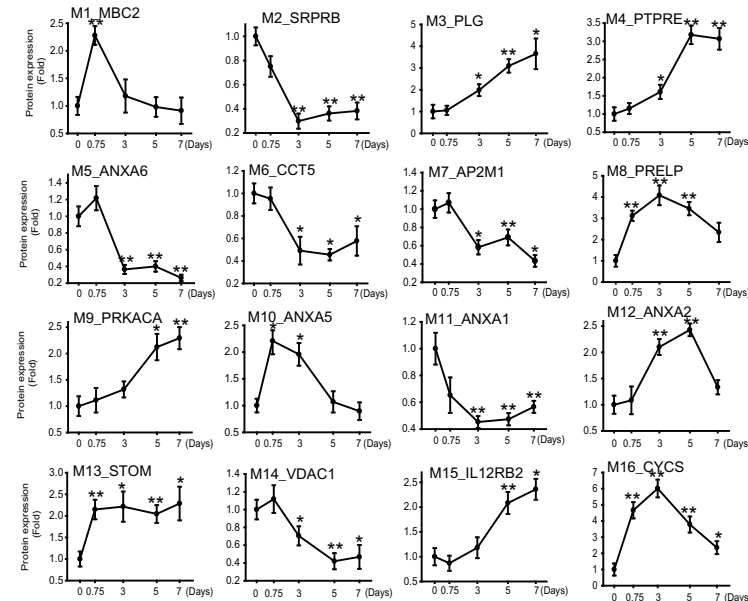

E

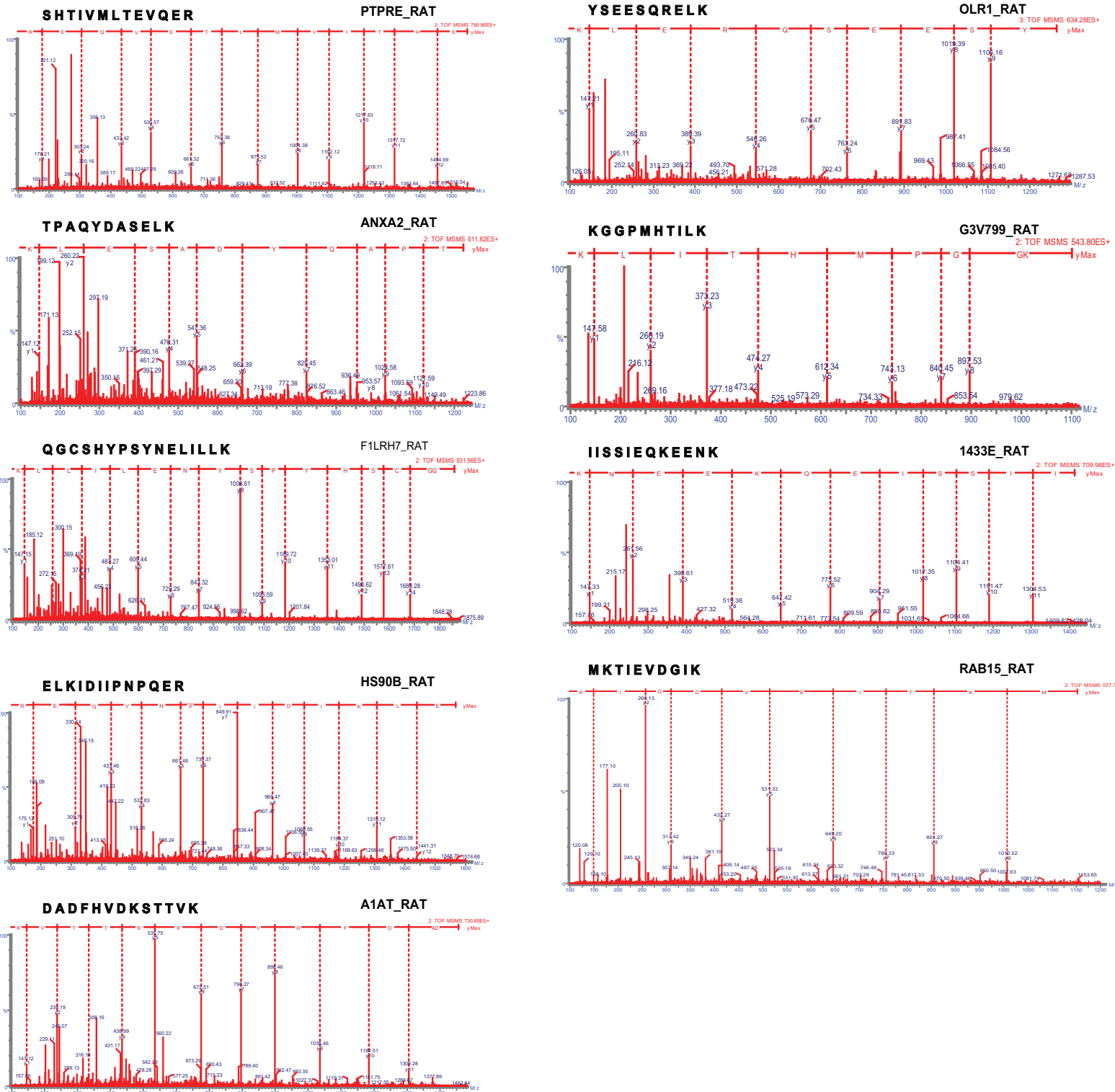

Supplement: S2 Fig — (A) Typical 2D-DIGE fluorescence images for S-100 cytosolic fraction. The protein samples from two consecutive time points were differently labeled with Cy3/Cy5 dyes. The internal standard (Std) was labeled with Cy2 dye. (B—D) Quantitative analyses of the differentially-expressed protein spots on 2D-DIGE gels. The Cy2/Cy3/Cy5 fluorescences were quantified using the DyCyer software tools in a Typhoon 9400 imager. The amount of Cy3/Cy5 fluorescence was normalized by that of Cy2 fluorescence and plotted in the histograms. Data in the histogram show the mean ± S.D. of the fold increase of the fluorescence intensities versus zero time point (n = 3 repeated experiments, *P<0.01, **P<0.001). The representative silver-stained 2D gels for cytosolic (B), nuclei (C), and membrane (D) fractions are shown. The position of selected protein spots with differential expression are indicated by arrows with serial spot numbers. The pH gradients and molecular weights are indicated on the horizontal and vertical axes, respectively. The graphs show. (E) LC-MS/MS spectra. The MS/MS spectrum of [M + H]+ ions of one of the peptides derived from the indicated protein is shown. PTPRE, Epsilon tyrosine phosphatase; ANXA2, Annexin A2; F1LRH7, IL-12 receptor beta 2; HS90B, Hsp90β; A1AT, Serine protease inhibitor 2c; OLR1, Oxidized LDL receptor-1; G3V799, Intima thickness-related receptor; 1433E, 14-3-3 epsilon; RAB15, Rab-15. (PDF) [file pone.0133845.s002.pdf]

**A**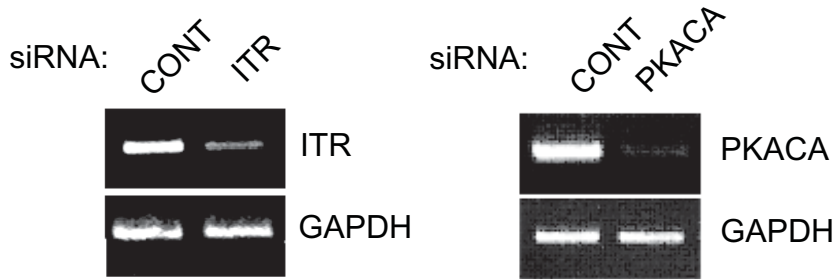**B**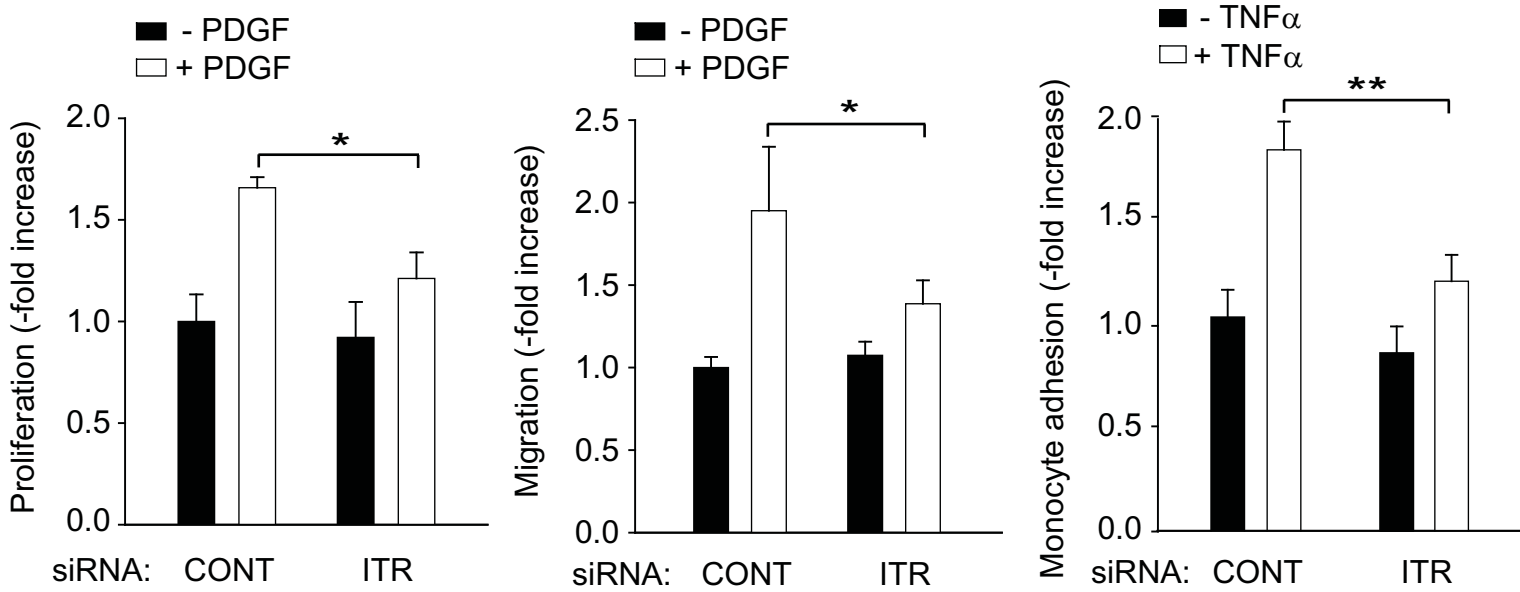**C**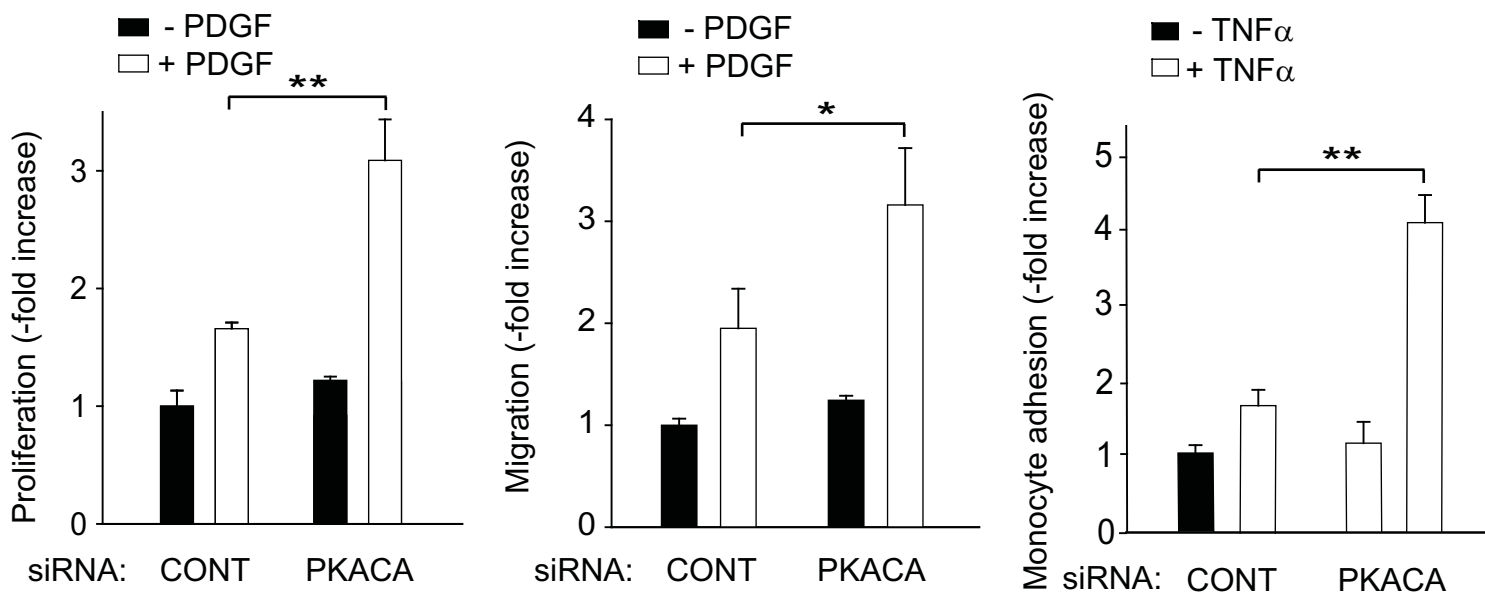

Supplement: S3 Fig — (A) Expression of ITR and PKACA mRNAs in HASMCs transfected with control or specific siRNAs. Total RNA was extracted from the HASMC cells using Trizol reagent (Invitrogen) and subjected to a reverse transcriptase (RT) reaction using ImProm-II reverse transcription system (Promega). The resulting cDNAs was amplified by PCR using Taq DNA polymerase (Promega) and the PCR products were visualized by agarose gel electrophoresis and ethidium bromide staining (BIPS system, Bio-Rad). (B) Negative effect of the ITR knockdown on PDGF-dependent SMC proliferation/migration and TNF-α-dependent monocyte adhesion. (C) Positive effect of the PKACA knockdown on PDGF-dependent SMC proliferation/migration and TNF-α-dependent monocyte adhesion. Each experiment was performed in triplicate and the data in the graph are means ± S.D. of fold increases from three independent experiments (*P<0.01, **P<0.005). (PDF) [file pone.0133845.s003.pdf]

**A**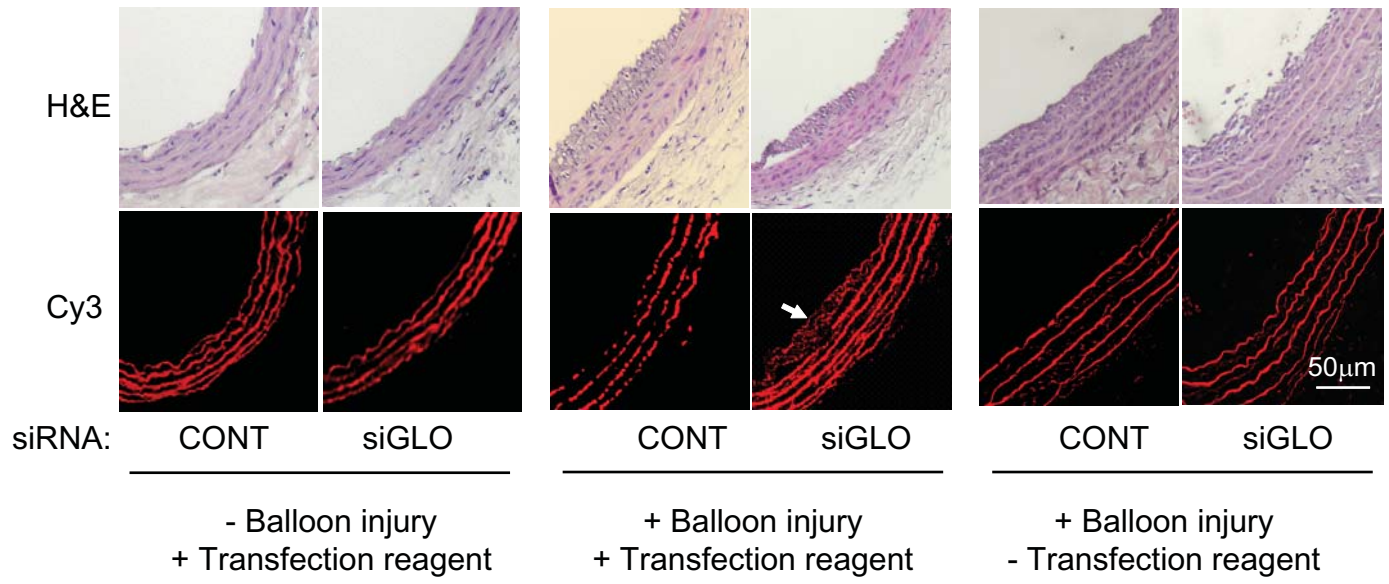**B**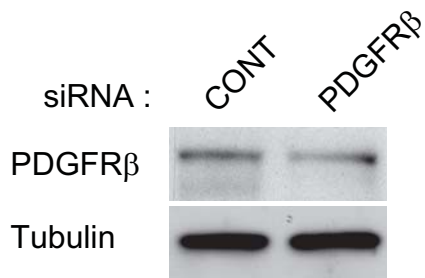**C**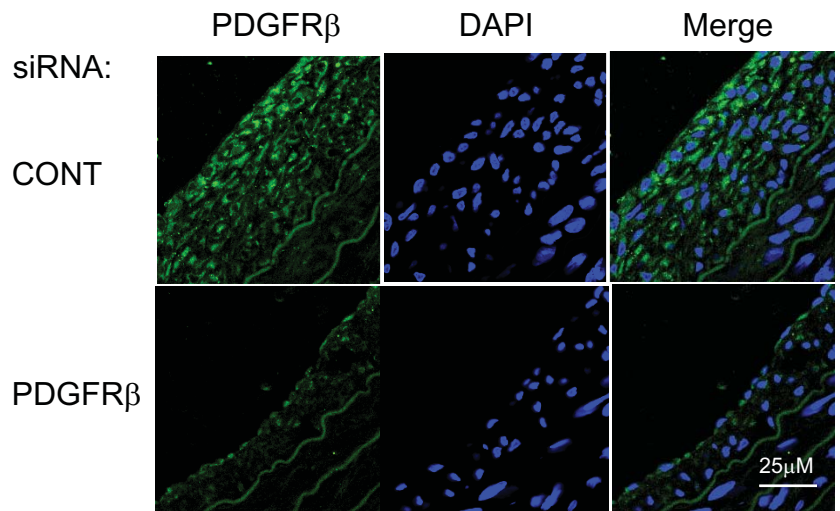**D**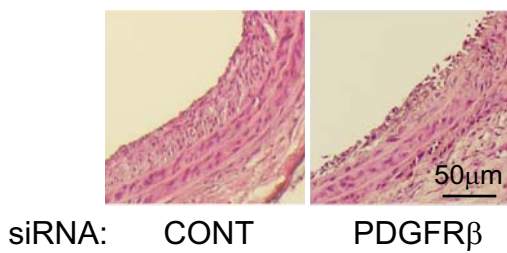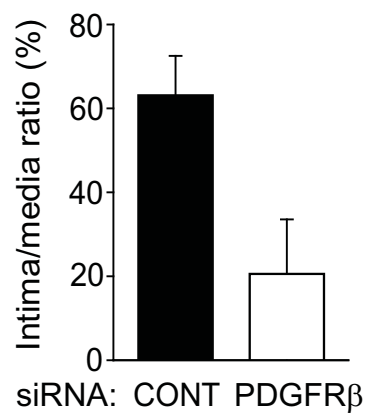

**F**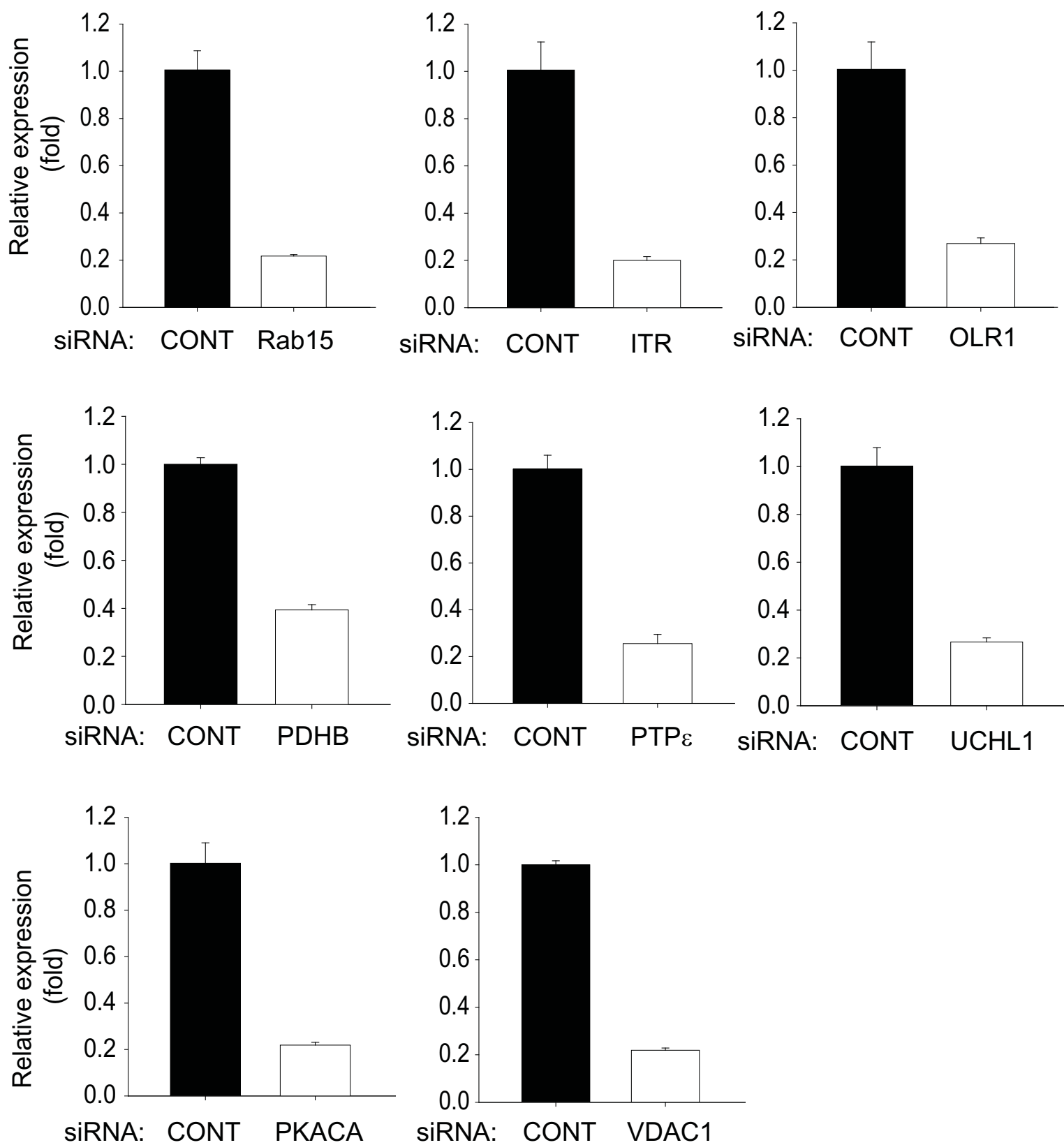

Supplement: S4 Fig — (A) In vivo siRNA transfection test. The fluorescent scrambled siRNA called siGLO (Dharmacon) was introduced into the lumen of normal or balloon-injured rat common carotid arteries with or without siPORT NeoFX transfection reagent. Note that the siGLO are transfected to the neointimal cells only when mixed with transfection reagent in the balloon-injured arteries. Arrow indicates the thickened neointimal layer. Representative HE-stained and fluorescence images from two independent experiments are shown. (B and C) In vivo transfection of rat PDGFRβ siRNAs sufficiently reduces the PDGFRβ expression in rat carotid arteries. The level of PDGFRβ was shown by immunoblotting (B) and immunofluorescence staining (C). (D) Neointimal thickening in the balloon-injured carotid artery is reduced by the PDGFRβ knockdown. Representative HE-stained images are shown. Data in the graph are means ± SEM of intima versus media ratio measured from HE-stained carotid samples (n = 3 rats per group). (E) Reduction of the target gene expression in the balloon-injured carotid arteries after the in vivo transfection of specific siRNAs. Total RNA from carotid vessels was purified using the RNeasy fibrous tissue kit (Qiagen). RNA (2 μg) was reverse transcribed using ImProm-II RT system (Promega). The real-time PCR was performed using specific primers in the presence of SYBR Green (Applied Biosystems) inside a fluorescent temperature cycler (ABI Prism 7000 sequence detection system, Applied Biosystems). The fluorescence signals were quantified by a comparative cycle threshold method. After finishing these cycles, a melting point was checked for specificity. The β-actin mRNA and 18S rRNA were used as endogenous reference genes. The expression level in the graph is means ± S.D. of fold changes relative to control-transfected samples (n = 3 rats per group). (PDF) [file pone.0133845.s004.pdf]

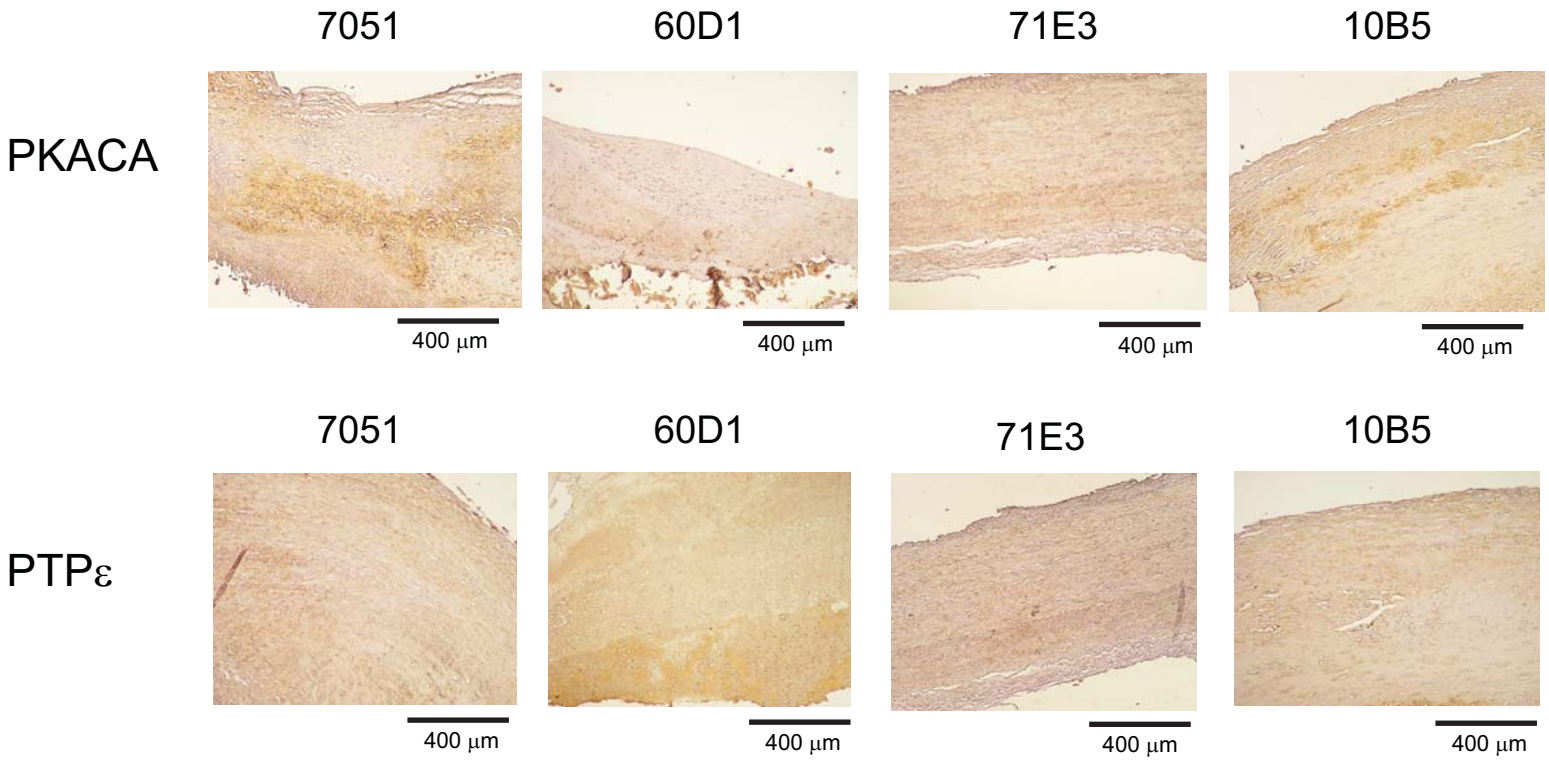

Supplement: S5 Fig — Immunohistochemistry was performed using the paraffin-embedded tissue sections of human carotid arteries with thickened intimal lesions (Origin Technologies, Rockville, MD, USA). The indicated proteins were stained with specific immunohistochemistry-compatible antibodies. The 3’,3’-diaminobenzidine (DAB)-stained images of carotid tissue sections are labeled with patient identification numbers. (PDF) [file pone.0133845.s005.pdf]
