# Supplementary material for: Vascular Proteomics Reveal Novel Proteins Involved in SMC Phenotypic Change: OLR1 as a SMC Receptor Regulating Proliferation and Inflammatory Response
Source: PLoS One. 2015 Aug 25;10(8):e0133845. doi: 10.1371/journal.pone.0133845 (PMC4548952; doi:10.1371/journal.pone.0133845)
Supplement: S1 Table — (PDF) [file pone.0133845.s006.pdf]

S1 Table

Spot	Gene ID	Protein name	SwissProt assession No.	MW(kDa)/pI	Coverage %	Mascot score	Matched peptides #	Unmatched peptides #	Identified method
S1	DNM1	Dynamin	O35303	83/6.64	20	77	12	52	PMF
S2	HSPCB	heat shock protein 90-beta	P34058	83/4.96	29	90	5		MS/MS
S3	AFP	alpha-fetoprotein	P02773	68/5.77	32	56	8	43	PMF
S4	CRMP1	Dihydropyrimidinase related protein	Q9JMG8	61/6.50	23	73	4		MS/MS
S5	GRP58	Protein disulfide-isomerase	P11598	56/5.88	60	74	12	31	PMF
S6	GC	Vitamin D-binding protein precursor	P04276	53/5.65	32	104	9		MS/MS
S7	ENO1	Enolase-1,alpha	P04764	47/6.16	38	86	7	42	PMF
S8	SERPINA1	serine protease inhibitor 2c	P17475	46/5.70	36	95	7		MS/MS
S9	PLD2	Phospholipase D2	F1LQD7	46/6.12	41	59	11	46	PMF
S10	GLUL	Glutamine synthase	P09606	42/6.64	48	178	13	54	PMF
S11	MDH1	Malate dehydrogenase-like enzyme	P04636	36/8.93	38	144	8	51	PMF
S12	LDHB	Lactate dehydrogenase B	P42123	37/5.70	41	112	12		MS/MS
S13	ALDOC	Aldolase C	P09117	39/6.67	36	146	11	74	PMF
S14	RAB3D	Rab-16	Q63942	24/4.75	27	78	8	94	PMF
S15	RAB15	Rab-15	P35289	24/5.36	27	82	5		MS/MS
S16	HSPB1	heat shock 27	P42930	23/6.12	27	69	5		MS/MS
S17	NSF	N-ethylmaleimide sensitive factor	O88960	17/5.57	32	84	6	68	PMF
S18	LGALS1	Galectin-1	P11762	15/5.09	34	59	7	52	PMF
N1	NEO1	Unnamed protein product	P97603	151/5.87	37	52	17	93	PMF
N2	LMNA	Lamin A	P48679	74/6.54	21	92	12		MS/MS
N3	GTF2F1	general transcription factor IIF polypeptide 1	Q6AY96	57/6.60	44	71	14	87	PMF
N4	SERPINF1	serine protease inhibitor,clade F	Q80ZA3	46/6.04	27	101	6	78	PMF
N5	OLR1	Oxidased low density lipoprotein receptor	O70156	42/6.05	14	59	4		MS/MS
N6	PDHB	Pyruvate dehydrogenase beta	P49432	39/6.20	17	64	5		MS/MS
N7	ITR	Intima thickness-related receptor	Q5XIJ2	38/6.45	19	57	4		MS/MS
N8	YWHAE	14-3-3 epsilon	P62260	29/4.63	25	73	5		MS/MS
N9	PCNA	PCNA	P04961	29/4.57	52	122	9	57	PMF
N10	UCHL1	Ubiquitin thiolesterase	Q00981	25/5.14	19	61	4		MS/MS
M1	MBC2	membrane bound C2 domain containing protein	Q9Z1X1	121/5.47	14	95	12		MS/MS
M2	SRPRB	Ba1-667	Q7TP24	107/8.35	20	85	10		MS/MS
M3	PLG	plasminogen	Q01177	91/6.79	27	129	18		MS/MS
M4	PTPRE	Epsilon tyrosine phosphatase	B2GV87	81/6.57	17	96	10		MS/MS
M5	ANXA6	Annexin A6	P48037	76/5.38	37	121	11		MS/MS
M6	CCT5	CCT(chaperonin containing TCP-1) epsilon subunit	Q68FQ0	59/5.50	59	72	19	67	PMF
M7	AP2M1	Adaptor protein complex AP-2,mul	P84092	49/9.57	27	106	8		MS/MS
M8	PRELP	PRELP	Q9EQP5	43/9.51	46	83	13	79	PMF
M9	PRKACA	PKA alpha catalytic subunit	P27791	41/8.70	31	87	9		MS/MS
M10	ANXA5	Annexin A5	P14668	36/4.91	38	56	7	52	PMF
M11	ANXA1	Lipocortin 1 (Annexin A1)	P07150	39/6.97	36	118	14		MS/MS
M12	ANXA2	Calpatin 1 heavy chain(annexin A2)	Q07936	39/7.55	61	81	12	55	PMF
M13	STOM	stomatin	Q5XI04	31/6.54	43	54	9	61	PMF
M14	VDAC1	VDAC-1	Q9Z2L0	31/8.62	34	72	6		MS/MS
M15	IL12RB2	IL-12 receptor beta 2	Q9Z0Z1	15/8.70	48	59	5		MS/MS
M16	CYCS	Cytocrome C,somatic	P62898	12/9.61	35	63	6	48	PMF
