# Supplementary material for: Vascular Proteomics Reveal Novel Proteins Involved in SMC Phenotypic Change: OLR1 as a SMC Receptor Regulating Proliferation and Inflammatory Response
Source: PLoS One. 2015 Aug 25;10(8):e0133845. doi: 10.1371/journal.pone.0133845 (PMC4548952; doi:10.1371/journal.pone.0133845)
Supplement: S2 Table — (PDF) [file pone.0133845.s007.pdf]

S2 Table

Fraction	Gene Symbol	Species	Locus ID	Accession	Catalog Number
**S1**	DNM1	h	1,759	NM_004408	M-003940-00
**S2**	HSPCB	h	3,326	NM_007355	M-005187-01
**S3**	AFP	h	174	NM_001134	M-011202-00
**S4**	CRMP1	h	1,400	NM_001313	M-009393-00
**S5**	GRP58	h	2,923	NM_005313	M-003674-00
**S6**	GC	h	2,638	NM_000583	M-011120-00
**S7**	ENO1	h	2,023	NM_001428	M-004034-00
**S8**	SERPINA1	h	5,265	NM_000295	M-008847-00
**S9**	PLD2	h	5,338	NM_002663	M-005064-00
**S10**	GLUL	h	2,752	NM_002065	M-008228-00
**S11**	MDH1	h	4,190	NM_005917	M-009264-00
**S12**	LDHB	h	3,945	NM_002300	M-009779-00
**S13**	ALDOC	h	230	NM_005165	M-012697-00
**S14**	RAB3D	h	9,545	NM_004283	M-010822-01
**S15**	RAB15	h	376,267	NM_198686	M-031564-00
		r	299,156	NM_198749	M-093352-01
**S16**	HSPB1	h	3,315	NM_001540	M-005269-01
**S17**	NSF	h	4,905	NM_006178	M-009401-00
**S18**	LGALS1	h	3,956	NM_002305	M-011718-00
**N1**	NEO1	h	4,756	NM_002499	M-009358-00
**N2**	LMNA	h	4,000	NM_170707	M-004978-00
**N3**	GTF2F1	h	2,962	NM_002096	M-019646-00
**N4**	SERPINF1	h	5,176	NM_002615	M-010153-00
**N5**	OLR1	h	4,973	NM_002543	M-003804-02
		r	140,914	NM_133306	M-093794-01
**N6**	PDHB	h	5,162	NM_000925	M-008803-00
		r	289,950	NM_001007620	M-086371-01
**N7**	ITR	h	160,897	NM_180989	M-019071-00
		r	306,165	NM_001006994	M-087817-01
**N8**	YWHAE	h	7,531	NM_006761	M-017302-00
**N9**	PCNA	h	5,111	NM_002592	M-003289-02
**N10**	UCHL1	h	7,345	NM_004181	M-004309-00
		r	29,545	NM_017237	M-089822-00
**M1**	MBC2	h	23,344	NM_015292	M-010652-00
**M3**	PLG	h	5,340	NM_000301	M-006001-02
**M4**	PTPRE	h	5,791	NM_006504	M-008068-02
		r	114,767	NM_053767	M-084709-01
**M5**	ANXA6	h	309	NM_001155	M-011210-00
**M6**	CCT5	h	22,948	NM_012073	M-012797-01
**M7**	AP2M1	h	1,173	NM_004068	M-008170-00
**M8**	PRELP	h	5,549	NM_002725	M-003693-00
**M9**	PRKACA	h	5,566	NM_002730	M-004649-00
		r	25,636	NM_001100922	M-093299-02
**M10**	ANXA5	h	308	NM_001154	M-011209-01
**M11**	ANXA1	h	301	NM_000700	M-011161-01
**M12**	ANXA2	h	302	NM_004039	M-010741-01
**M13**	STOM	h	2,040	NM_004099	M-016971-00
**M14**	VDAC1	h	7,416	NM_003374	M-019764-00
		r	83,529	NM_031353	M-096517-01
**M15**	IL12RB2	h	3,595	NM_001559	M-007932-00
**M16**	CYCS	h	54,205	NM_018947	M-017355-00
